# A Deployable 4D Printed, Mucoadhesive and Magnetically Guided Patch for Local Therapy of Gastric Cancer

**DOI:** 10.1002/adhm.202501085

**Published:** 2025-07-26

**Authors:** Dina B. Mahmoud, Martin Börner, Christian Wölk, Michaela Schulz‐Siegmund

**Affiliations:** ^1^ Pharmaceutical Technology Institute of Pharmacy Faculty of Medicine Leipzig University 04317 Leipzig Germany; ^2^ Department of Pharmaceutics Egyptian Drug Authority, formerly known as National organization for Drug Control and Research Giza Cairo Egypt; ^3^ Institute for Inorganic Chemistry Leipzig University 04103 Leipzig Germany

**Keywords:** 3D printing, hydrogel, magnetic biomaterials, mucoadhesive material, shape actuation

## Abstract

A new perspective for treatment of gastric cancer utilizing a four‐dimensional (4D) printed design for deployable and magnetically guided local drug releasing patches is proposed. The deployed patches are envisioned to adhere at the tumor site in order to locally deliver high drug amounts to the tissue. The approach is considered as a platform for a gastric patch design that is flexible with reference to size and shape, active pharmaceutical ingredient (API) and dosages as well as providing unidirectional drug release. Agarose is employed as main component for the patch for its water‐actuated shape transformation (curling‐shrinkage/deployment) capabilities. This patch can be compactly deformed upon drying for easy ingestion. The patch is envisioned to rehydrate and expand in the stomach. In order to promote mucoadhesion, agarose hydrogel is modified with chitosan and included SPIONs to allow for magnetic guidance. The study focuses on the design and fabrication process, physicochemical characterization, and magnetization evaluation of the composite hydrogel. The hydrogel is used as an ink for 3D printing and processed into a patch loaded with a model drug. By leveraging the combination of 4D printing, magnetic guidance, and localized drug delivery, this patch has the potential to improve the effectiveness of stomach cancer treatments.

## Graphical abstract

A new perspective for treatment of gastric cancer is proposed utilizing 4D printing to develop shape morphing, deployable, and magnetically guided local drug releasing patches. The deployed patches are envisioned to adhere at the tumor site to locally deliver high drug amounts. The approach represents a platform for a gastric patch design fulfilling the personalized medicine approach.

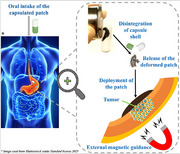

## 1 Introduction

Gastric cancer is ranked among the leading causes of cancer‐related mortalities. It is accounted for over one million new cases and ≈769 000 deaths (1 of 13 death cases) worldwide in 2020, making it the fifth most common cancer and the fourth leading cause of cancer death.^[^ ^1^ ^]^ The high mortality rate associated with stomach cancer is largely due to the asymptomatic nature in early stages, leading to late diagnosis, and limited treatment options.^[^ ^2^ ^]^ Conventional treatments include surgery, chemotherapy and radiation therapy.^[^ ^3^ ^]^ Typically, chemotherapy is the first‐line treatment strategy for advanced stages of gastric cancer. Further, perioperative (neoadjuvant) chemotherapy, postoperative (adjuvant) chemotherapy or chemoradiation are listed as preferred strategies in current treatment guidelines for localized disease.^[^ ^4^ ^]^ However, the major drawback of systemic chemotherapy is the lack of specific toxicity as well as the difficulty to achieve high drug concentrations at the tumor site. Consequently, higher systemic drug doses are required to effectively reach tumors which causes undesirable systemic side effects.^[^ ^5^ ^]^ Moreover, the intravenous administration of chemotherapy involves multiple hospitalizations which may reduce patient compliance.^[^ ^4^, ^6^ ^]^ These challenges emphasize the urgent need for innovative treatment strategies which provide local delivery of anticancer drugs to the tumor site^[^ ^7^ ^]^ with minimal systemic toxicity. Unfortunately, due to the harsh environment of the stomach, positioning such a local delivery system at the tumor site is challenging. Recently, Bandi and Venuganti have reported for the first time the fabrication of polymeric gastric patches to treat gastric cancer^[^ ^8^, ^9^ ^]^ utilizing conventional casting method. Interestingly, 3D printing technology, if utilized for patch fabrication, can permit flexible customization, quick prototyping and precise dose control.^[^ ^10^, ^11^ ^]^ Thus, patches of different surface areas and shapes can be quickly developed. Recently, 3D printing of deployable patches has gained a particular interest in material and biomaterial sciences. Aaron et al. have fabricated a hydration‐based actuatable composite patch consisting of agarose hydrogel and a 3D printed poly(caprolactone) scaffold which guides the folding of agarose during dehydration and rehydration.^[^ ^12^ ^]^ Based on agarose folding, another study has proposed a foldable pill enclosing a magnet for localizing the patch to be used as a physical plug on gastric perforation.^[^ ^13^ ^]^ The dynamic and stimuli responsive 3D printed materials have been lately recognized as 4D printed materials due to their shape transformation capabilities counting the time as the fourth dimension.^[^ ^14^ ^]^ There are a limited number of 4D printing pharmaceutical and biomedical applications proposed in literature such as gastroretentive, intravesical and esophageal drug delivery systems.^[^ ^15^ ^]^ The shape morphing ability of the 4D printing materials can be utilized to deliver large drug delivery systems with minimum invasion through narrow body orifices or to extend their residence time at the site of action.^[^ ^16^ ^]^ To the best of our knowledge, no 4D printed hydrogel gastric patch for local drug delivery to gastric tumors is reported in literature. Herein, we report the development of a 4D printed, magnetically guided, water‐actuated shape changeable composite hydrogel patch that is positioned using minimally invasive procedures and retained within close proximity to a gastric tumor. This patch is proposed as a local treatment strategy to achieve high drug concentrations at the tumors’ sites which, in turn, has the potential to decrease systemic toxicity. This approach employs 4D printing to create a highly customized and effective treatment platform to fit each patient's need and the geometry of different sized tumors. The shape changing capability of the patches facilitates their noninvasive easy delivery through a capsule. Upon reaching the stomach, the patch will rehydrate, expand, and regain its original large shape, adhering to the gastric mucosa for localized and sustained drug release. To enhance the therapeutic efficacy and specificity of the patch, a novel enteric adhesive polymer coating is simply applied as a backing layer conferring unidirectional drug release properties to the patch. Additionally, the hydrogel patch is further augmented with superparamagnetic iron oxide nanoparticles (SPIONs) which enable external manipulation using a magnetic field^[^ ^17^, ^18^ ^]^ providing a noninvasive means of positioning the patch precisely at the tumor site. Besides, SPIONs containing patches may be employed for eradication of the adjacent tumors,^[^ ^19^ ^]^ via magnetic hyperthermia. This dual functionality, as a drug delivery system and SPIONs carrier, presents this hydrogel patch as a versatile platform and powerful complementary tool to manage stomach cancer. Further, the 3D printing‐manufacturing process presents our patch as a tool for implementing the next generation of personalized therapeutic strategies which allows “on‐demand” production of dosage forms and changing the concept of “one medicine fits all”.^[^ ^20^ ^]^ Recently, *p‐Coumaric acid* (*p*‐CoA) has shown to exhibit an anticancer activity against gastric cancer,^[^ ^21^, ^22^, ^23^, ^24^ ^]^ thus it has been employed in this study as a model drug for loading our patch.

## 2 Materials and Methods

### 2.1 Materials

Eudragit FL 30 D‐55 was obtained as a gift from Evonik, Germany. Agarose, Chitosan (low molecular weight), Dulbecco's Modified Eagle's Medium‐High glucose, penicillin/streptomycin, fetal bovine serum (FBS) and DMSO were purchased from Sigma‐Aldrich. Pepsin was purchased from Panreac Applichem, Germany. Ferric chloride hexahydrate and ferrous sulfate hepta‐hydrate were purchased from Merck, Germany. Disc magnet (Nickel (Ni‐Cu‐Ni), 30 mm diameter, 10 mm height, 20 kg holding force) was purchased from Supermagnete, Germany. Simulated gastric fluid (SGF) was prepared with 2 g NaCl in 1L deionized water, pH was adjusted to 1.2 with 10 M HCl and then 3.2 g pepsin was added. Further, acetate buffer pH 4.5 was used to represents middle fed state simulated gastric fluid (FeSSGF). Caco‐2 cell line was obtained from DMSZ‐German Collection of Microorganisms and cell cultures GmbH (DSMZ no.: ACC 169).

### 2.2 Formulation of SPIONs

Amounts of ferric chloride hexahydrate (2.34 g) and ferrous sulfate hepta hydrate (1.38 g) (molar ratio 1.75:1) were weighed and added in a round bottom flask to 100 mL deionized water and stirred with an overhead paddle stirrer at 200 rpm under nitrogen atmosphere for 1 h at 70 °C; and then 100 mL ammonia solution (25%) was added gradually with stirring at 500 rpm at 70 °C for 1 h. The nanoparticles were collected with a magnet. The SPIONs were washed 3 times with hot water and dried overnight in an oven at 50 °C.

### 2.3 Characterization of the Formulated SPIONs

#### 2.3.1 Particle Size, Polydispersity Index, and Zeta Potential

The size of the collected SPIONs was determined directly after preparation before and after drying. To this end, the dried SPIONs were redispersed in water, with sonication, in a concentration of 0.6 µg mL^−1^ and then the particle size, polydispersity index and zeta potential values of the samples were recorded using dynamic light scattering technique (Litesizer, Anton Paar, Graz, Austria).

#### 2.3.2 Preparation of the Composite Hydrogels

**Figure 1:**
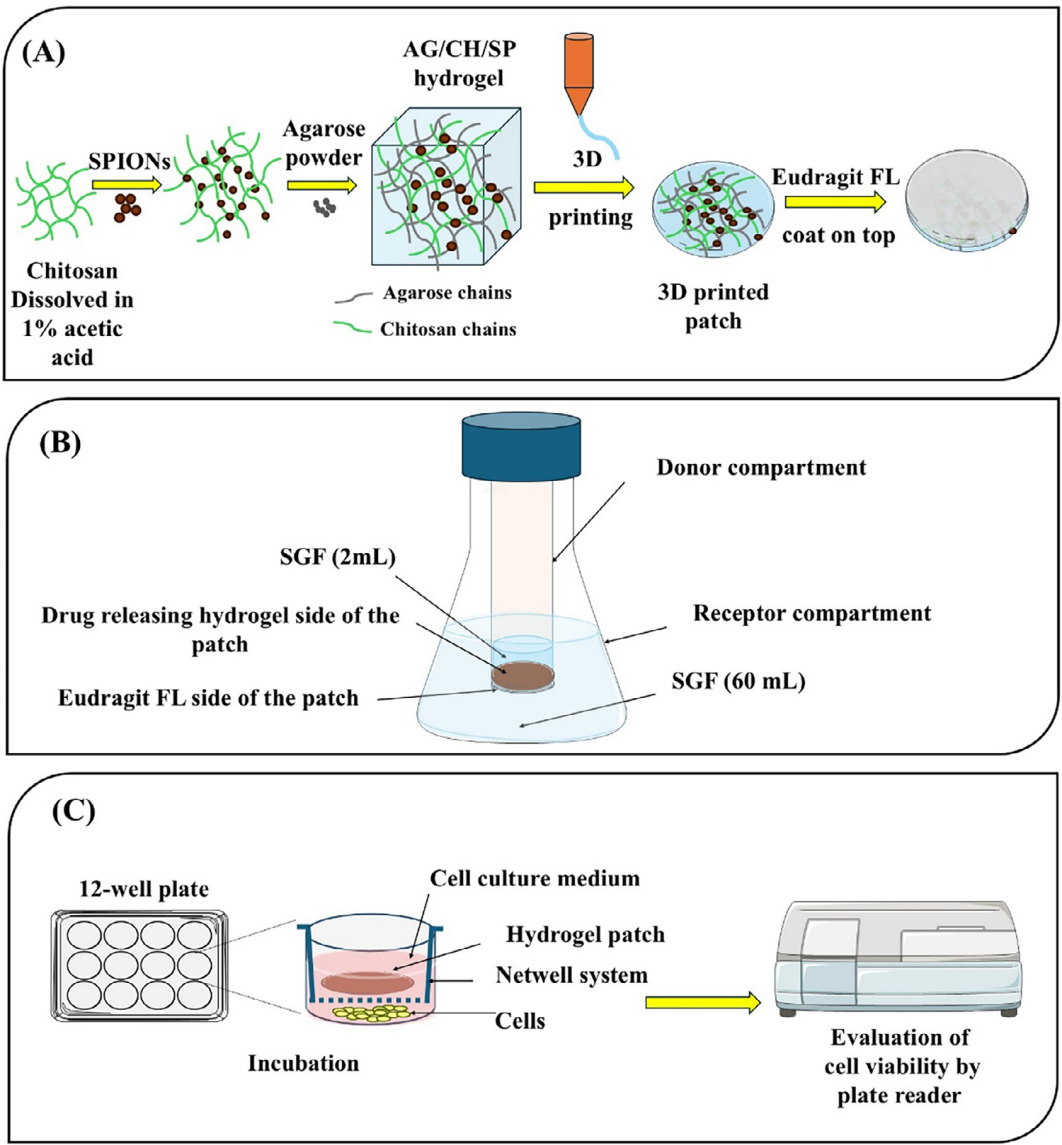
Schematic diagram illustrates: A) The preparation of AG/CH/SP composite hydrogel patch, B) in vitro release experiment set up to test the functionality of Eudragit FL enteric coating of the patch, and C) cytotoxicity study in which treating Caco‐2 cells were treated with the patch placed in the Netwell system to prevent cell damage due to physical contact with the patch.

A composite hydrogel (AG/CH/SP, **Figure** **1**A) composed of 3% agarose (main actuatable polymer), 1% chitosan (mucoadhesive polymer) and 0.6% SPIONs (magnetically actuatable material) was prepared as follows: Chitosan powder was dissolved in 1% acetic acid with stirring at 400 rpm to yield a concentration of 2% w/w. Chitosan solution (5 g) was mixed with 0.15 g 0.05 m sodium hydroxide solution (to adjust pH to 5), 2 g dispersion of SPIONs in water (30 mg g^−1^) and 0.3 g agarose powder; and then the weight was completed to 10 g with deionized water. The mixture was stirred in water bath (80 °C) for 30 min till a homogenous ink was obtained. Additionally, pure agarose (AG) hydrogel (3%), 3%/1% agarose/chitosan composite hydrogel gel (AG/CH), and 3%/0.6% agarose/SPIONs hydrogel (AG/SP) hydrogels were also prepared for characterization studies.

### 2.4 Rheological Evaluation

Rheological study has been conducted using Physica MCR 301 rheometer, Anton Paar GmbH, Graz, Austria. Temperature sweep test has been done by decreasing the temperature of the heated hydrogels from 80 to 20 °C, at a frequency of 1 Hz and Amplitude gamma = 1%. Storage modulus (G′) and loss modulus (G″) were recorded. Sol‐gel temperature was generated for each hydrogel as a crossover point of G′ and G″ curves. To ensure that the hydrogels do not liquefy if reheated to body temperature, we increased the temperature of the formed hydrogels from 20 to 50 °C at a frequency of 1 Hz and Amplitude gamma = 1% in an additional temperature sweep. G′ and G″ were recorded. Additionally, a flow ramp test was performed at 73 °C (printing temperature) over a logarithmic shear rate range from 0.01 to 2000 s⁻¹ to evaluate the shear thinning behavior of the formulations. Viscosity profiles were generated and results were further fitted to Power‐law model using this equation:^[^ ^25^ ^]^

(1) $$\tau =K\cdot \gamma^{\cdot n}$$

where *τ* is the shear stress (Pa), K is the consistency coefficient (Pa·s), γ̇ is the shear rate (s^−1^) and n is the Power‐law index that is used to judge the flow behavior as when n < 1, the fluid is shear‐thinning, n  = 1 the fluid is Newtonian, and n  > 1, the fluid is shear‐thickening.

Further, yield point was determined by evaluating the flow curve using Bingham model according to this equation:^[^ ^25^, ^26^ ^]^

(2) $$\tau =\tau_{B}+\eta_{B}\cdot \dot{\gamma }$$

where τ is shear stress (Pa), γ̇ is shear rate (s^−1^), τ_B_  is the Bingham yield point (Pa) and η_B_ is the Bingham viscosity (Pa.s).

#### 2.4.1 Fourier‐Transform Infrared Spectroscopy (FTIR)

FTIR analysis was performed for agarose, chitosan, SPIONs and the dried hydrogel samples in the range of 4000–400 cm^−1^ using Bruker Alpha II FTIR spectrometer that was equipped with a Platinum ATR and operated with OPUS software.

#### 2.4.2 Differential Scanning Calorimetry (DSC)

DSC study was performed using hydrogel samples that were dried at room temperature for 48 h. Samples (5 mg) of agarose, chitosan, SPIONs and the dried hydrogel samples were weighed in 40 µL Al‐crucibles and the measurements were done in a temperature range of 25 to 400 °C (10 K min^−1^) under nitrogen atmosphere utilizing Mettler Toledo DSC equipped with STARe Software 14.0, Switzerland.

#### 2.4.3 Thermogravimetric Analysis (TGA)

The thermal performance of agarose, chitosan, SPIONs and the dried hydrogel samples were examined by thermogravimetric analyzer (TGA/DSC1 system with STARe Software 14.0, Mettler Toledo, Switzerland) in the temperature range of 25 to 800 °C (10 K min^−1^) and nitrogen flow rate of 50 mL min^−1^.

### 2.5 Investigation of the Magnetization Properties of the Ink

The magnetic measurements were performed at powdered samples using a MPMS 7XL SQUID magnetometer (Quantum Design). Magnetization curves were measured at 310 K with fields up to 70 kOe. Field cooled (FC) and Zero field cooled (ZFC) curves were recorded between 2–300 K with 0.5 kOe external field applied during the measurements.

#### 2.5.1 Scanning Electron Microscopy (SEM)

Imaging AG/CH/SP hydrogel samples were done by SEM before and after incubation with SGF with pepsin for 72 h. The samples were allowed to dry at ambient temperature for 24 h to remove residual moisture, before scanning them by Phenom XL Desktop SEM (Thermo Fisher Scientific) with an accelerating voltage of 10 KV. Images were captured at different magnification (from 1000 x to 20000 x) using a backscattered electron detector (BSD Full mode). Energy Dispersive X Ray (EDX) mapping analysis was performed to identify and visualize the distribution of elements (C, N, O, and Fe) present in the sample at certain spots.

### 2.6 Printability and Shape Fidelity of the AG/CH/SP Ink

RegenHu extrusion 3D printer was used for the printing process. BioCAD software was utilized to design the patches and to generate G codes. Screening of needle diameter, printing temperature, extrusion pressure and layer thickness was done. Different feed rates were tested, photographs were taken and the filament diameters were determined for each feed rate using image J software. The shape fidelity was assessed using a planar structure that predominantly extends along two directions (*x* and *y* plane). We used images of a printed grid pattern to measure the filament diameter at different locations to assess filament uniformity expressed as coefficient of variation (CV%). Further, the spreading ratios were determined by normalizing the printed filament diameters that were measured to the needle diameter,^[^ ^27^ ^]^ at different feeding rates. Further, the printability index (*P*_r_) was calculated from the perimeter and area of the pores in the grid pattern using the following formula.^[^ ^28^ ^]^

(3) $$P_{r}=\frac{\;L^{2}}{16A}$$

where *L* is the perimeter and A is the area.

Additionally, geometrical accuracy was assessed by comparing the dimensions of the printed structure to the computer‐aided design (CAD) of a 4‐layered flower like design.

### 2.7 Water‐Actuated Shape Transformation of the Patches

#### 2.7.1 Effect of the Drying Conditions on the Shape Transformation

Square patches (20 mm) of 3 layers with 0.7 mm line spacing were 3D printed using AG/CH/SP hydrogel ink. The patches were dried either overnight on a mesh at room temperature or in an oven at 84 °C for 2 h. Then the dried patches were rehydrated in SGF containing pepsin (pH 1.2). The weights and dimensions of the patches were recorded at 5, 10, 20, 30, and 60 min; the swelling indices (SI) were calculated from this equation and plotted against time with Wd: weight of the dried sample, Ws: weight of the swollen sample.

(4) $$\mathrm{SI}=\frac{\mathrm{Ws}-\mathrm{Wd}}{\mathrm{Wd}}$$

#### 2.7.2 Effect of Rehydration Medium on the Shape Transformation

Square patches (20 mm) of 3 layers with 0.5 mm line spacing were 3D printed using AG/CH/SP hydrogel ink. The 3D printed patches were dried overnight on a mesh at room temperature. Then the dried patches were rehydrated either in SGF (pH 1.2) or FeSSGF (pH 4.5). The weights and dimensions of the patches were recorded at 5, 10, 20, 30, and 60 min; the SI values were determined and plotted against time.

#### 2.7.3 Effect of Layers Number Or Line Spacing on the Shape Transformation

Patches of 3 layers and others of 5 layers (0.5 mm line spacing) were 3D printed and dried overnight on a mesh at room temperature. Then the dried patches were rehydrated in SGF (pH 1.2). The weights and dimensions of the patches were recorded at 5, 10, 20, 30, and 60 min; the SI values were determined and plotted against time. Patches of 3 layers with line spacing of either 0.5 or 0.7 were also investigated.

#### 2.7.4 Effect of Entering Coating on Shape Transformation

In order to achieve unidirectional drug release, enteric coating of one side of the patches was performed. Patches of 5 layers of AG/CH/SP hydrogel were 3D printed, aliquots of 35 µL of Eudragit FL 30 D‐55 dispersion were cast over one side of the patch placed on glass slides and the slides were tilted to ensure the spread of Eudragit FL on the patch. The patches were kept in the oven at 40 °C for 15 min to allow drying of Eudragit FL coat and then the patches were inverted on the other side and dried overnight at room temperature on a mesh where Eudragit FL layer was kept on the bottom side. The dried patches were rehydrated in SGF (pH 1.2). The weights and dimensions of the patches were recorded at 5, 10, 20, 30, and 60 min; the SI values were determined and plotted against time.

### 2.8 Preparation of *p*‐CoA Loaded Hydrogel

*p*‐CoA was used as a model drug to study the drug release and the effectiveness of the enteric coating process. The hydrogel ink was prepared as mentioned before and the drug was incorporated. The final composition of the hydrogel was 3% Agarose, 1% chitosan, 0.6% SPIONs, 1% *p*‐CoA.

### 2.9 Physicochemical Characterization of *p*‐CoA Loaded Hydrogel

*p*‐CoA loaded hydrogel was characterized by assessment of rheological properties, DSC and FTIR measurement as previously mentioned for the plain hydrogels.

### 2.10 Assay of Drug Content

Samples of 3D printed patches were crushed, added to 3 mL ethanol and agitated for 24 h at 200 rpm. After that, the samples were centrifuged and *p*‐CoA content was quantified by measuring the absorbance of the supernatants at 290 nm. Calibration curve of *p*‐CoA was generated in ethanol and used for assay calculations. % Drug content was estimated from the following equation:^[^ ^29^ ^]^

(5) $$\;\%\mathrm{Drug}\;\mathrm{content}=\frac{\mathrm{Actual}\;\mathrm{drug}\;\mathrm{amount}}{\mathrm{Theoretical}\;\mathrm{drug}\;\mathrm{amount}}\times 100\%$$

### 2.11 In Vitro Drug Release and Functionality of the Enteric Coat

*p*‐CoA was used as a model drug to study the drug release and the effectiveness of the enteric coating process. To test the effect of printing design on the release of *p*‐CoA from the patches, 4 designs of the patches were prepared. Patches were either 3D printed with 3 or 5 layers (L) utilizing line spacing (LS) of either 0.5 or 0.7 mm and then coated with Eudragit FL prior to drying at room temperature. The release of *p*‐CoA from the patches was tested by placing the patches in 60 mL SGF (at 37 °C, 50 rpm). In order to judge the functionality of the enteric coat of the backing side, the 3D printed *p*‐CoA loaded patches were prepared with Eudragit FL layer as mentioned earlier. The patches were mounted between a donor compartment that was filled with 2 mL of SGF and then the compartment was immersed in an acceptor compartment, where 60 mL of SGF was added (Figure 1B). Three patches were mounted in a way that allows the entering backing side to face the acceptor medium. The study was conducted in 37 °C with shaking at 50 rpm, the apparatus was covered to prevent evaporation of the medium. Samples of 1 mL were withdrawn at predetermined time intervals at 0.5, 1, 2, 4, 8, and 24 h and replaced with fresh medium. The absorbance of the samples was measured using plate reader at 290 nm and quantified against calibration curve of *p*‐CoA in SGF. The cumulative % *p*‐CoA released was determined and plotted versus time.

### 2.12 Ex vivo Mucoadhesion

The stomach of freshly sacrificed cow was obtained from the butcher's house and rinsed with phosphate buffer saline (pH 7.4) to remove chyme. The printed patches were immersed in SGF for 30 min and then they were glued to a metal disc shaped holder with a diameter of 12.5 mm. The stomach tissue was cut into a rounded shape and also adhered to another metal holder which was fixed to the tissue rig holder of EZ Test Texture Analyzer (Shimadzu, Germany). The patch holder was fixed in the upper probe of the instrument in a way that allows the patch to face the mucosal side of the stomach tissue. The patch was allowed to contact the stomach tissue for 1 min. After that, the probe was withdrawn at a speed of 1 mm/min. The peak force require to detach the patch from the stomach tissue was attained from the force displacement curve. Besides, the work of adhesion was determined as the integral of the resultant force distance profiles. The strength of mucoadhesion was investigated for the drug‐loaded patches, free‐drug patches with or without chitosan and the backing layer side.

### 2.13 Assessment of Tensile Strength of the Patches

The mechanical properties of the *p*‐CoA loaded AG/CH/SP patches with or without backing layer were studied using a tensile testing instrument (EZ Test Texture Analyzer, Shimadzu, Germany) to determine their ultimate tensile strength. The 3D printed patches (5L/0.5LS) of 15 mm diameter were prepared, dried and rehydrated in SGF for 6 h. The patches were fixed in the instrument holder and the probe was set to move upward in a speed of 0.5 mm/min The ultimate tensile strength was recorded as the maximum stress point in the stress versus strain curve.

### 2.14 Ex vivo Drug Permeability

Ex vivo permeation study was performed utilizing freshly excised cow stomach tissue obtained from butcher's house. The tissue was cut into circular pieces with a diameter of 32 mm and each piece was mounted between the donor compartment, that contained 3 mL SGF with Pepsin, where *p*‐CoA‐loaded patches were hydrated. The stomach tissue was mounted in a way that allows the mucosal side to face the donor medium. The donor compartment was then immersed in the acceptor compartment which contains 20 mL SGF with pepsin. The medium was maintained at 37 °C and agitated at 50 rpm for 24 h. 1 mL aliquots from the acceptor compartment were withdrawn at 1, 2, 4,8, and 24 h and replaced with fresh medium. The withdrawn samples were analyzed spectrophotometrically for *p*‐CoA content at 290 nm using standard calibration curve of *p*‐CoA. Further, at the end of the experiment, in order to determine the remaining *p*‐CoA in the patch and in the stomach tissue, the patches were removed and extracted in 3 mL ethanol. In addition, the stomach tissue was soaked in 10 mL ethanol and kept for 24 h with stirring at 200 rpm and then the samples were centrifuged at 17500 rpm for 5 min at 5 °C and the amount of *p*‐COA was quantified in the supernatant.

### 2.15 Cytotoxicity

The cytotoxicity of the formulated plain polymeric patches and *p*‐CoA loaded patches were assessed using Caco‐2 cells. We used a 12 well plate with Netwell insert polystyrene mesh (15 mm, mesh size of 74 µm), to hold the patches above the cells to prevent any physical contact induced cell damage (Figure 1C). Cells were seeded in a density of 3 × 10^5^ cells per well with a 2 mL DMEM high glucose medium supplemented with 10% fetal bovine serum, 1% NEAA, 1% Penicillin/streptomycin^[^ ^30^ ^]^ in a 5% CO_2_ atmosphere at 37 °C for 24 h. On the next day, the medium was replaced with a fresh 2 mL medium and the cells were treated with *p‐*CoA, *p‐*CoA loaded AG/CH/SP, plain AG/CH/SP, AG/CH patches for further 24 h, so that the final *p*‐CoA concentration is 10 mM in each well. Finally, the cell culture medium was removed in each well and replaced with 500 µL fresh medium plus 50 µL Rottitest Vital reagent. The cells were incubated for 1 h at 37 °C and 5% CO_2_ and then the absorbance of the samples was recorded at 450 nm using plate reader (Synergy H1, Biotek, Bad Friedrichshall, Germany). The % cell viability was calculated using to the following equation, where the control group represents Caco‐2 cells treated only with the medium:

(6) $$\%\;\mathrm{cell}\;\mathrm{viability}=\frac{\text{OD 450 nm of the sample}}{\text{OD 450 nm of the control}}\times 100\%$$

### 2.16 Statistical Analysis

Statistical analysis was performed using OriginPro2019 software (Northampton, Massachusetts, USA). Data pre‐processing was done utilizing Shapiro‐Wilk test for testing normality; outliers were judged using Grubbs test and excluded only when technically justified. For comparisons between two groups, a two‐sided Student's t‐test assuming equal variances was used. The assumption of equal variance was assessed using an F‐test, and Welch's correction was applied when variances were unequal. In case where normality assumption was not met, the nonparametric Mann–Whitney U test was used instead of *t*‐test. One way ANOVA with Tukey post hoc test was applied for means comparison between more than two groups. Analysis was done using a significance level of *p* < 0.05 that was set as a minimal level of statistical significance. More stringent levels (e.g., *p* < 0.01) are reported where applicable. Data are presented as mean ± standard deviation (SD), sample sizes (n) and the statistical tests applied are specified in the corresponding figure legends. For rheological measurements, when plotted on a logarithmic scale, only the upper SD (+SD) is shown for clarity.

## 3 Results and Discussion

### 3.1 Formulation and Characterization of SPIONs

With the aim to generate a magnetically‐guided drug delivery system, which permits site specific transport and localization,^[^ ^31^, ^32^ ^]^ we first produced SPIONs and then mixed them with chitosan solution and agarose to form SPIONs loaded composite hydrogels. We used an alkaline co‐precipitation method as it is cost effective, simple and rapid. In this method, the magnetic particles are formed when the pH of an oxygen‐free medium containing Fe^2+^ and Fe^3+^ is raised to alkaline values.^[^ ^33^ ^]^ SPIONs were successfully prepared and collected with a magnet. Before drying the particle size of the SPIONs was 292 ± 21 nm (0.24 ± 0.02 PDI) while the dried and subsequently redispersed SPIONs showed a larger size of 558 ± 80 nm with polydispersity index of 0.25 ± 0.006 determined by dynamic light scattering. Further, SPIONs exhibited a zeta potential value of −31.6 ± 4.4 and −22.4 ± 1.9 mV before and after drying respectively. The increase in particles size suggests an initial formation of magnetic nanocrystals that aggregated to superparamagnetic particles during processing. Magnetic aggregates have been reported to form in various sizes ranging from tens of nanometers up to few micrometers due to assembly of primary superparamagnetic crystals (less than 25 nm in size).^[^ ^34^ ^]^ In proportion to their volume, aggregates provide increasing magnetic response^[^ ^35^ ^]^ and improved shelf‐life.^[^ ^34^ ^]^ Overall, the presence of SPIONs in the form of aggregates was advantageous here, since we needed to maximize the superparamagnetic properties of the nanoparticles and embed them homogeneously in a hydrogel matrix. Furthermore, a larger size would reduce diffusion from the local patch to prevent unwanted systemic absorption of the nanoparticles.

### 3.2 Rheological Evaluation

**Figure 2:**
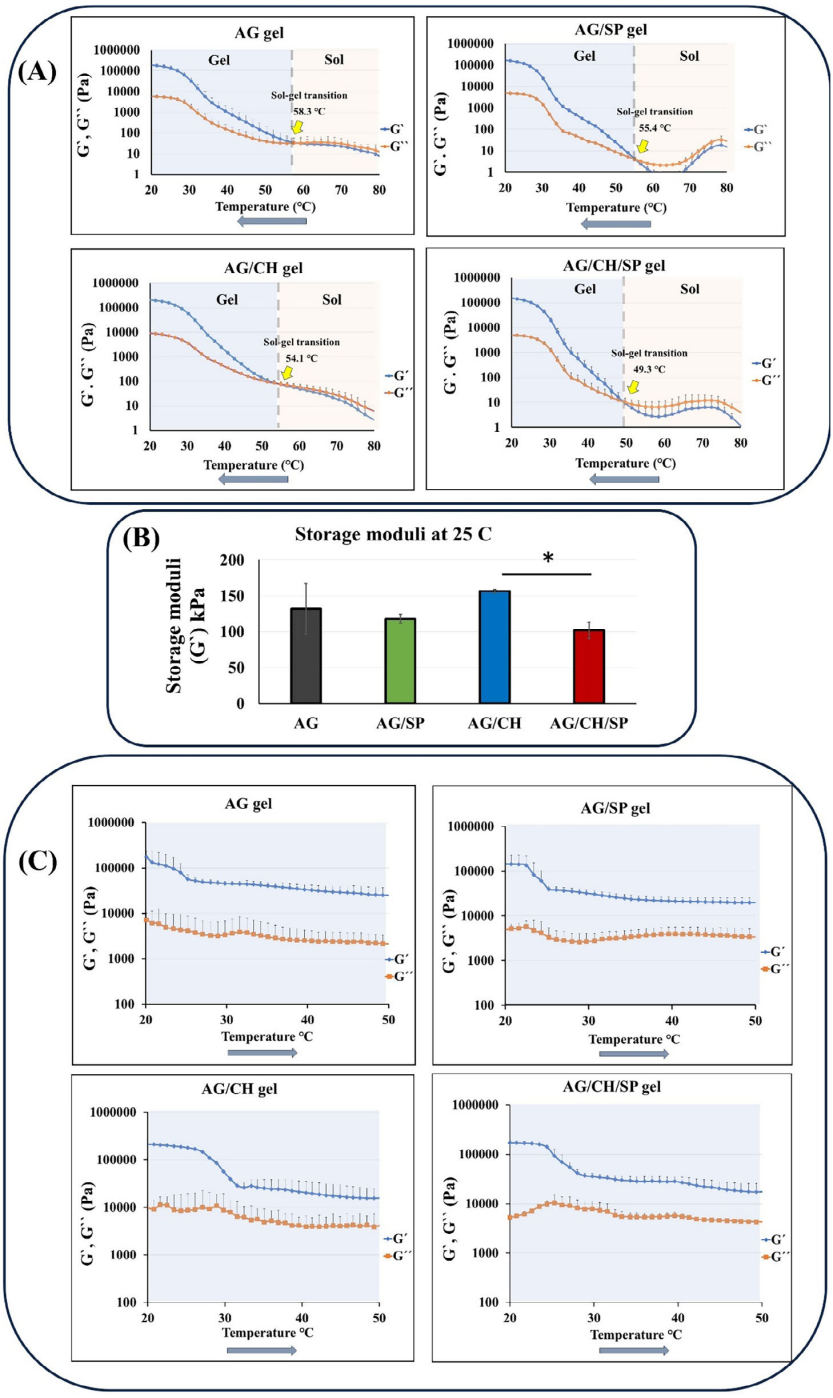
Rheological evaluation of the composite hydrogels. A) Storage (G′) and loss (G″) moduli of the prepared composite hydrogels determined via temperature sweep tests from 80 to 20 °C (cooling rate was 5 °C min^−1^), the yellow arrows indicate sol‐gel transition temperatures of the hydrogels determined as the crossover point of the storage and loss moduli curves (n = 3–7, for clarity, error bars represent +SD due to logarithmic scale). B) Storage moduli of the hydrogels at 25 °C (n = 3–7). (*) indicates significance difference (*p* < 0.05), one‐way ANOVA with Tukey post hoc test (C) Storage and loss moduli curves determined upon heating from 20 to 60 °C (5 °C min^−1^). (n = 3, for clarity, error bars represent +SD due to logarithmic scale).

Thermoresponsive polymeric hydrogels generally depict rapid transformation from viscous liquid state to crosslinked stable hydrogel state once they reach a critical temperature. Thermoresponsive polymers, exhibiting temperature dependent sol‐gel transition, are categorized by a critical solution temperature below or above which they gel or liquify. Pluronics, for example, show a lower critical solution temperature (LCST) forming a gel above the LCST, whereas agarose shows an upper critical solution temperature (UCST) like behavior, forming a gel as the temperature decreases due to hydrogen bonding and the formation of double helices.^[^ ^36^, ^37^ ^]^ **Figure** **2** shows the rheograms of the prepared agarose and composite hydrogels. All the formulated composite hydrogels exhibited thermoresponsive sol‐gel transition properties switching from sol to gel upon cooling in the conducted temperature sweep test (Figure 2A). This behavior is crucial for printing the hydrogels by extrusion. Upon extrusion 3D printing of the composite hydrogel, the composite material needs to be maintained in a sol state at an elevated temperature to permit smooth and continuous extrusion through the nozzle without needle clogging. Once the temperature drops upon deposition on a cooler substrate the material transforms to the gel state solidifying the printed structure. This temperature induced sol‐gel transition ensures that the printed structures maintain their integrity without the need for additional crosslinking or curing approaches (e.g., chemical or photo‐crosslinking). In order to determine the printing conditions for each composite hydrogel, the crossover points between the storage and loss moduli curves were recorded as the sol‐gel transition temperature (Figure 2A). The sol‐gel transition temperatures ranged from 49.3 °C for AG/CH/SP to 58.3 °C for the pure agarose gels. A combination of chitosan and SPIONs decreased the sol‐gel transition temperature of the agarose hydrogel even stronger than the single components. The values of the storage moduli of the hydrogels recorded at 25 °C (as it reflects the status post printing and on shelf) are shown in Figure 2B. The addition of chitosan or SPIONs did not significantly affect the storage modulus of agarose gel. Addition of SPIONs to AG/CH gel, however, significantly decreased the storage modulus (*p* < 0.05), which may be explained by the interruption of the gel network by SPIONs. Nevertheless, all formed gels exhibited storage moduli higher than 100 kPa. Further, to fulfill the requirements of a stable patch, it was critical to ensure that the gels would not convert to sol state after oral administration at body temperature at 37 °C. Figure 1C indicates the thermal stability of the formed gels, as the storage and loss moduli curves did not overlapped upon raising the temperature from 20 to 60 °C. Hence, the thermoresponsive sol‐gel transition behavior of the composite hydrogel is important in this work for processing of the patches, once they are printed it remains in the gel state retaining the functional stability and shape fidelity post‐printing at room or body temperature. The deformation and shape recovery of the 3D printed hydrogel patch, however, are triggered by swelling and drying (water‐induced actuation) and not by thermal actuation. Generally, when extrusion 3D printing is intended, a material should exhibit a shear thinning behavior decreasing its viscosity when exposed to high shear stress (during nozzle extrusion).^[^ ^38^ ^]^ As shown in Figure S1 and Table S1 (Supporting Information). all hydrogels exhibited shear‐thinning behavior (n < 1) at 73 °C signifying their extrudability. AG/CH composite exhibited lower flow index (0.62) and significantly increased the yield stress to 69.4 Pa, indicating that the incorporation of chitosan enhanced structural integrity and exhibited pronounced shear‐thinning. AG/CH/SP composite combined high yield stress (67.1 Pa) and moderate shear‐thinning, suggesting optimal printability and shape fidelity.

### 3.3 FTIR

**Figure 3:**
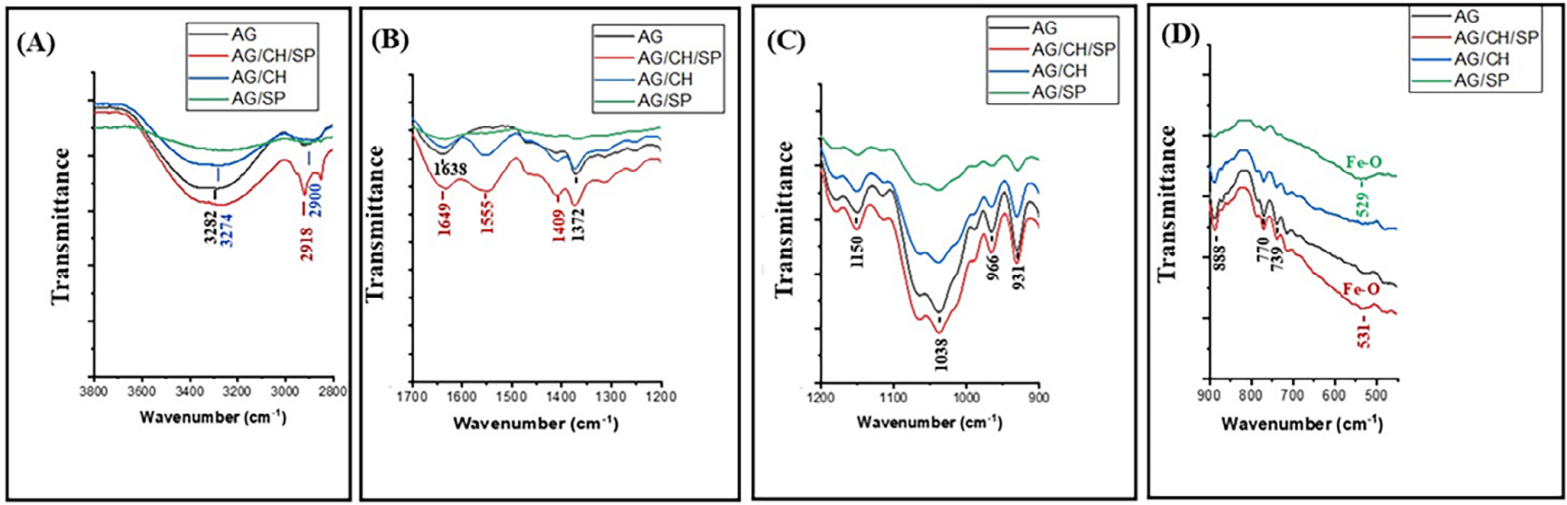
FTIR spectra of AG, AG/SP, AG/CH, and AG/CH/SP hydrogels.

FTIR spectra of powdered agarose, chitosan and SPIONs are depicted in Figure S2 (Supporting Information) and of AG, AG/SP, AG/CH, AG/CH/SP hydrogels in **Figure** **3**A–D. The FTIR spectrum of the AG gel depicts a broad peak at 3000–3700 cm^−1^ (Figure 3A) referring to the stretching vibration of O─H group.^[^ ^39^ ^]^ Besides, the bands of 3,6‐anhydrogalactose bending at 770 and 931 cm^−1^ and the stretching vibration band of C─O at 1038 cm^−1^ are also observable.^[^ ^40^ ^]^ The bands at 1372 and 2918 cm^−1^ (Figure 3A,B) are assigned to asymmetric bending and stretching vibration of ‐CH_2_, respectively.^[^ ^41^ ^]^ The intensity of these bands decreased in the AG/SP hydrogel suggesting strong interaction between agarose and SPIONs. Also, the observed decrease in intensity of OH stretching and OH bending vibration that is seen in the spectrum of AG/SP gel suggest interaction between agarose and SPIONs disrupting polymer water hydrogen bonding (Figure 3A). Further, the spectrum of pure SPIONs shows vibration band of Fe─O bond at 539 cm^−1[^ ^42^ ^]^ (Figure S2, Supporting Information), which has been red shifted to lower wavenumber 529 cm^−1^ in AG/SP (Figure 3D). This also suggests interaction of SPIONs with agarose through hydrogen bond formation (Fe─O to Fe─OH) complying with previous report which demonstrated that the formation of Fe─OH produces red shift in stretching vibration of Fe─O bond.^[^ ^43^ ^]^ The absorption band at 3422 cm^−1^ in the spectrum of SPIONs (Figure S2, Supporting Information) represents the stretching vibration of hydroxyl groups connecting to SPIONs surface which enhance the tendency of agglomeration of SPIONs.^[^ ^44^ ^]^ The spectrum of chitosan exhibits (Figure S2, Supporting Information) the characteristic absorption band of amide I (C═O stretching) and amide II (N–H bending) at 1649 and 1555cm^−1^, respectively.^[^ ^45^, ^46^ ^]^ These bands appear broader in case of AG/CH and AG/CH/SP gels (Figure 3B) suggesting the formation of hydrogen bond with agarose^[^ ^47^ ^]^ and/or SPIONs. In the spectrum of AG/CH/SP gel, the characteristic Fe─O band still can be observed (531 cm^−1^). No clear decrease in intensities of the characteristic bands of agarose as observed for AG/SP were found in AG/CH/SP gels, which suggest that, in presence of both agarose and chitosan, SPIONs favor interaction with chitosan. This interaction is also suggested from the appearance of CH_2_ stretching vibration modes at 2918 and 2850 cm^−1^ in AG/CH/SP hydrogel.^[^ ^48^ ^]^ Further, this is clearly visible from the changes in the stretching vibration band of C‐O of agarose at 1038 cm^−1^ (Figure 3C). The intensity of this band decreased in both AG/CH and AG/SP spectra suggesting interaction of agarose with either chitosan or SPIONs, while this is not the case in AG/CH/SP spectrum indicating that chitosan and SPIONs favor their interaction together leaving the agarose C─O band unaffected.

### 3.4 DSC

Results of DSC analysis of the hydrogel mixtures are depicted in Figure S3A (Supporting Information). We observed distinct water evaporation patterns, providing insights into the water‐polymer interactions and their implications for shape actuation. AG hydrogel exhibited a broad endothermic peak between ≈58–162 °C, indicative of a gradual evaporation process associated with both free and bound water. This heterogeneous distribution of water within the hydrogel, with free, loosely bound and tightly bound water evaporating differentially^[^ ^49^ ^]^ justifies the water‐responsive actuation of agarose, where bound water contributes to polymer chain flexibility and their gradual diffusion allows for more controlled and tunable shape change rather than abrupt shrinkage. In contrast, AG/SP composite hydrogel displayed a sharp endothermic peak around ≈69–133 °C, corresponding to less bound water which can rapidly evaporate from the hydrogel. This is likely due to the interaction between SPIONs and agarose OH groups, which disrupts hydrogen bonding with water. Interestingly, the incorporation of chitosan into AG/SP hydrogel restored a broader DSC peak (≈57–176 °C). This indicates an increased proportion of bound water in AG/CH/SP hydrogel, probably due to the preferential interaction of SPIONs with chitosan as suggested by FTIR permitting agarose‐water interaction. The enhanced water retention capacity of this composite hydrogel suggests gradual and controlled water loss, promoting improved flexibility and reversible shape change upon dehydration and rehydration. Further, an exothermic peak at ≈ 222 °C is observed immediately after water evaporation in AG/CH/SP hydrogel suggesting crystallization or structure reorganization of the composite matrix. This observations emphasize the importance of controlling the drying process for reversible shape actuation.

### 3.5 TGA

Thermogravimetric analysis of pure agarose hydrogels and the composite hydrogels are displayed in Figure S3B,C (Supporting Information). TGA results are in agreement with DSC data, for instance, AG/SP hydrogel depicted a distinctive weight loss step due to water desorption at midpoint temperature of ≈106 °C, which is clearly lower than in case of AG hydrogel (≈127 °C), AG/CH (131 °C) and AG/CH/SP (≈119 °C) which exhibited gradual weight loss steps. This suggests that the water is more loosely bound in AG/SP hydrogel due to interaction between agarose and SPIONs. This resulted in a more rigid hydrogel network as suggested by TGA curves which exhibit weight loss steps due to polymeric chain decomposition of AG/SP hydrogel at broader temperature range than AG indicating higher thermal stability and crosslinking degree upon SPIONs incorporation to AG hydrogels. Overall, all the hydrogels depict a major weight loss step corresponding to polymeric chain decomposition at onset temperatures higher than 181 °C. Our results reveal that there is no degradation of the polymeric chains of the hydrogels at the potential printing temperature.

Overall, the FTIR, DSC, and TGA findings supported the selection of AG/CH/SP hydrogel for water‐responsive shape actuation, as the presence of bound water enables switchable flexibility, while the polymer network maintains structural integrity for shape recovery. Though chitosan addition was intended initially to improve mucoadhesion of the hydrogel, results suggests its beneficial role for preserving shape actuation of agarose in presence of SPIONs.

### 3.6 Investigation of the Magnetization Properties of SPIONs and SPIONs Loaded Ink

**Figure 4:**
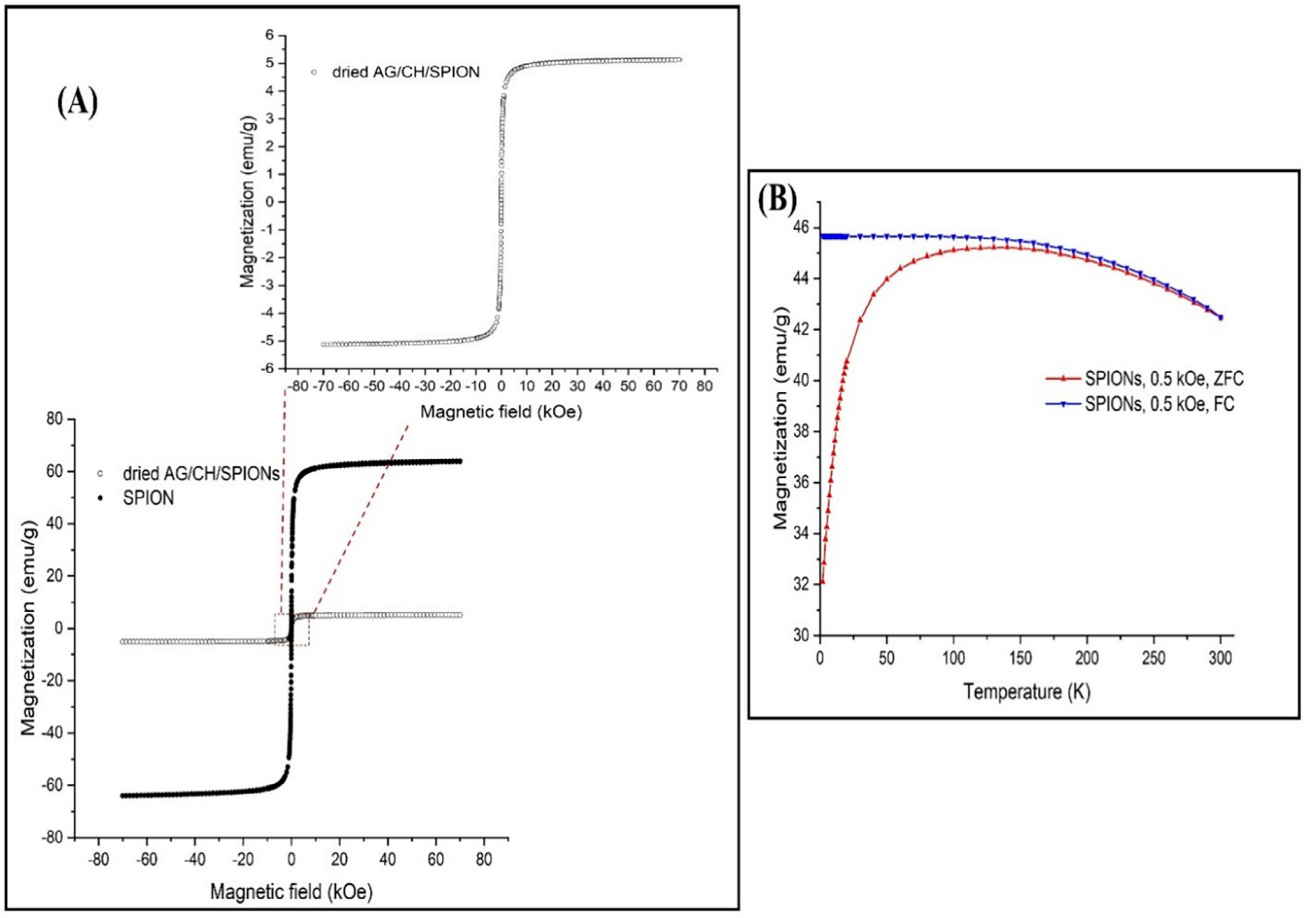
A) Magnetization curves of both SPIONs and dried AG/CH/SP at 36.85 °C (310 K) in the range of −70 to 70 kOe. B) Temperature‐dependent magnetization curves between 2 and 300 K with an external field of 0.5 kOe.

The magnetic behavior of the formulated SPIONs and AG/CH/SP hydrogel ink was investigated by SQUID magnetometry. **Figure** **4**A depicts the magnetization curves of SPIONs and AG/CH/SP at body temperature (36.85 °C). The saturation magnetization of pure SPIONs is around 64 emu/g. This result is in compliance with previous reports,^[^ ^50^, ^51^ ^]^ and higher than the reported values of SPIONs in other studies (≈38, 42.5, 57.5, and 58 emu g^−1^),^[^ ^52^, ^53^, ^54^, ^55^ ^]^ which reported on smaller size nanoparticles (10–20 nm). The saturation magnetization declines to ≈5 emu/g when SPIONs were incorporated in the hydrogel. This is attributed to the reduced concentration of SPIONs with their entrapment in the hydrogel matrix. Nevertheless, the saturation magnetization of the hydrogel is in compliance with previous reports for biomedical applications.^[^ ^56^, ^57^ ^]^ The magnetization curves revealed that both, the nanoparticles and the hydrogel, show superparamagnetic behavior with negligible remanence and coercivity. Superparamagnetism is the capability of being magnetized responding to an application of magnetic field without remanence (permanent magnetization) after the removal of the magnetic field.^[^ ^44^ ^]^ This behavior signifies the potential use of our system in various biomedical applications, for instance drug delivery,^[^ ^58^ ^]^ magnetic resonance imaging (MRI)^[^ ^59^ ^]^ and magnetically targeted cancer therapeutics.^[^ ^60^ ^]^ The FC and (ZFC curves were recorded under an external magnetic field of 0.5 kOe in order to evaluate the temperature‐dependent magnetic behavior of the SPIONs. The ZFC curve provides a continuous increase of the magnetization to ≈45 emu/g at 140 K (−133 °C) related to the progressive unblocking of the magnetic moments with rising temperature and the resulting alignment with the external magnetic field. The subsequent drop to 42.5 emu g^−1^ at 300 K (27 °C) is a consequence of the rising thermal energy allowing the spins to alter their direction of magnetization freely. At elevated temperatures, ZFC and FC curves superimpose. Below a certain temperature a splitting of both curves is observed and the FC curve maintains a magnetization of ≈45 emu g^−1^ at 2 K.

#### 3.6.1 SEM

**Figure 5:**
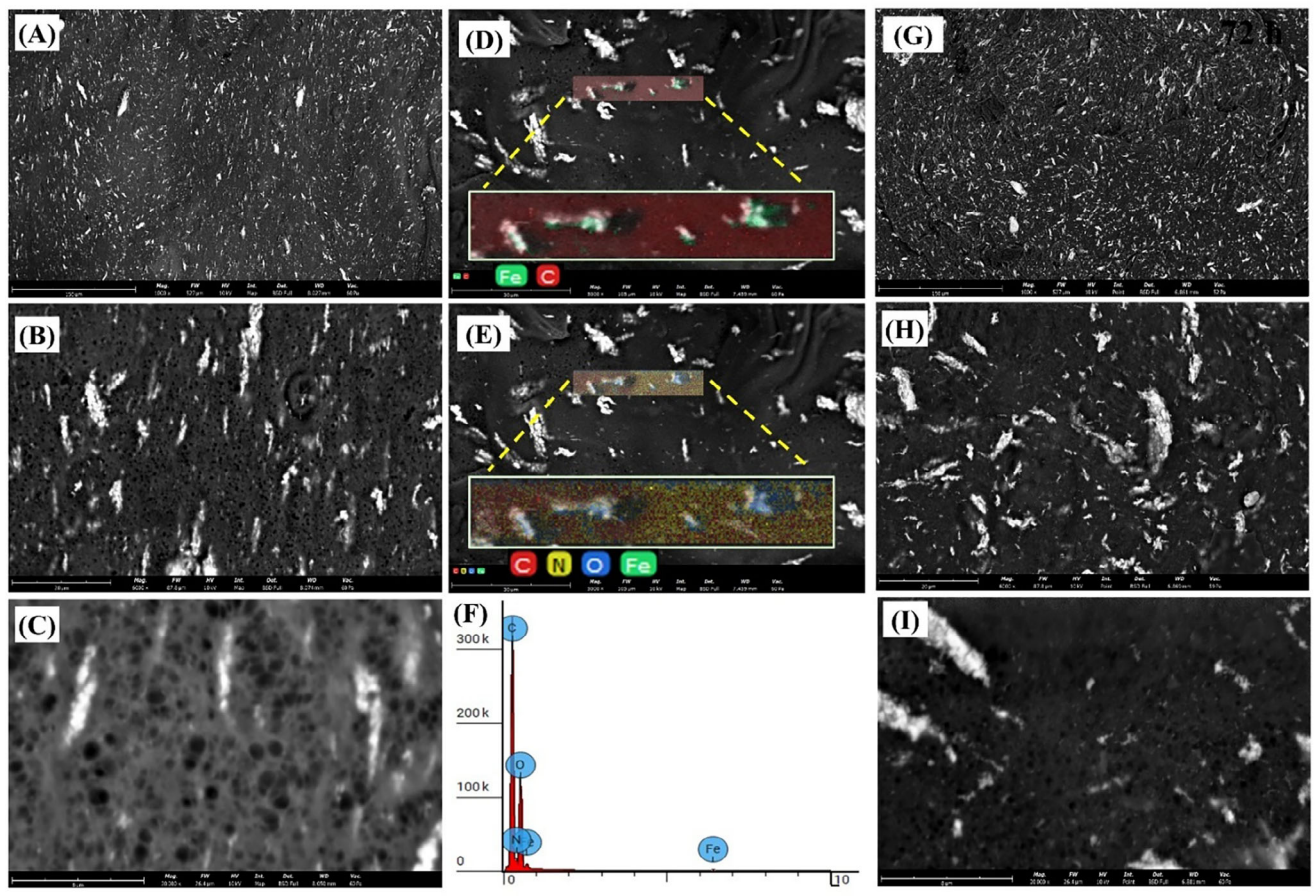
SEM images of dried samples of AG/CH/SP hydrogel at various magnifications: A) 1000, B) 6000, and C) 20 000; EDX mapping analysis of D) Fe and C. E) C, N, O, and Fe. F) EDX Spectrum depicts the distribution of C, N, O, and Fe. Images of dried samples of AG/CH/SP hydrogel after incubation with SGF for 72 h at various magnifications: G) 1000, H) 6000, and I) 20 000 confirming the retention of SPIONs in the hydrogel matrix.

SEM images are depicted in **Figure** **5** which exhibit the presence of SPIONs as brighter spots. They appear more intense under backscattered electron detection due to the higher atomic number of iron compared to the surrounding polymer matrix. Further, EDX mapping analysis confirm the presence of iron and oxygen in these brighter spots (Figure 5D–F). Moreover, the retention of SPIONs in the gel matrix after incubation of samples in SGF with pepsin for 72 h is noticeable as shown in Figure 5G–I. As mentioned earlier, the larger size of SPIONs and their presence in form of aggregates minimized their diffusion out of the hydrogel preserving the magnetic properties of the system.

### 3.7 Printability and Shape Fidelity of the AG/CH/SP Ink

The concept of this study was to formulate an ink in order to print a drug releasing patch that can be magnetically guided and at the same time exhibit mucoadhesive properties. To this end, we fabricated a thermoresponsive composite hydrogel ink consisting of agarose combined with chitosan and SPIONs to enhance the mucoadhesion of the hydrogel and impart superparamagnetic properties. Needle 23, with an internal diameter of 0.33 mm was selected for printing AG/CH/SP ink. The syringe was maintained at a temperature (73 ± 1 °C) above the sol‐gel temperature of the gel to avoid needle clogging. External air pressure was set at 3 bar while the internal air pressure in the printhead was kept at 0.1 bar. The layer thickness was set to be 0.3 mm.

**Figure 6:**
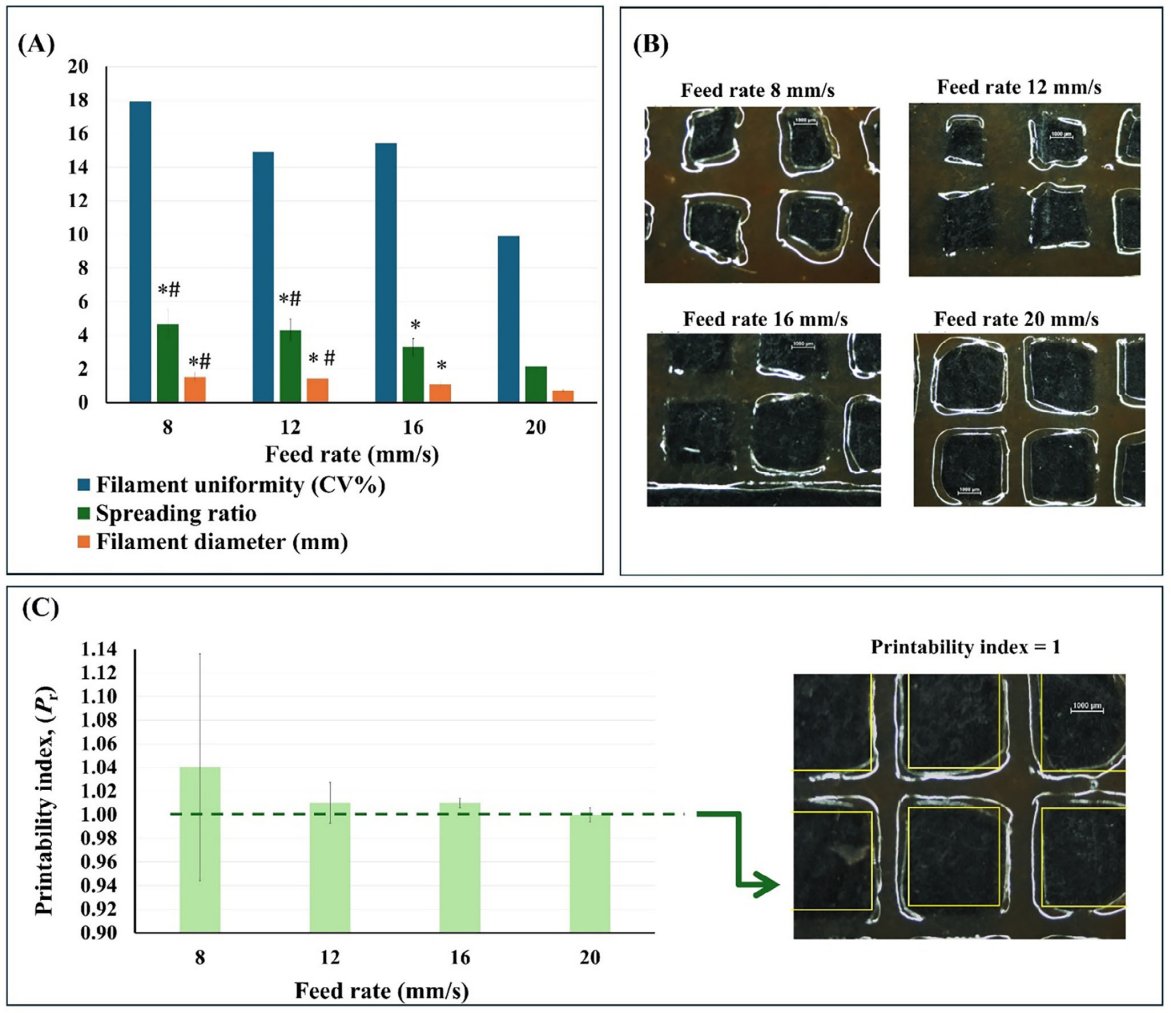
Assessment of shape fidelity of AG/CH/SP hydrogel ink. A) Filament uniformity expressed as % CV, spreading ratio and the printed filament diameter. Results determined by measuring filament width at different locations in the grid pattern, n = 12–24. *, # indicates significant difference from feed rate 20 or 16 mm s^−1^, respectively, determined by one way ANOVA followed by Tukey test. B) Pictures showing the grid pattern. C) Printability index depending on the printing feed rates (n = 8).

Results of shape fidelity assessment revealed that increasing feed rate from 8 to 20 mm/s produces more uniform filaments with lower CV (9.9%), lower spreading ratio (2.2 ± 0.2) and hence smaller diameters (0.71 ± 0.07 mm) (**Figure** **6**A,B). Generally, spreading ratio between 1 and 3 are acceptable for printing.^[^ ^61^, ^62^ ^]^ Further, filament stacking and deformation due to surface tension can be detected from the filament width at the intersection points of two filaments,^[^ ^63^ ^]^ where fusion induces diameter increase in *x*–*y* plane upon filament relaxation and spreading over the underlying layer. As shown in Figure S4A (Supporting Information); Figure 6B, printing at feed rate 20 mm s^−1^ produce minimal filament stacking at the intersections. Additionally, the *P*_r_ which was determined from the perimeter and area of the pores in the grid pattern is commonly used to judge the printability; as the ideal axial pores in a 0–90 grid pattern exhibit as squares in *x*–*y* plane. In such a case, high geometric accuracy can be judged as the *P*_r_ equals 1 while *P*_r_ < 1 or *P*_r_ > 1 indicates more round or irregular shaped pore geometries, respectively. The results revealed that printing with feed rate 20 mm/s yielded *P*_r_ of 1 (Figure 6C) confirming excellent fidelity in planar features. Hence, a feed rate of 20 mm s^−1^ was chosen for further printing. Moreover, we assessed the shape fidelity of a 3D printed flower like patches composed of 4 intersecting elliptical layers (Figure S4B, Supporting Information) to evaluate the geometrical accuracy as well as the fusion height and layer stacking by comparing the geometries at certain points and the theoretical height of the construct designed in the CAD file with the obtained one. Interestingly, this shape can investigate the problem of some printers in achieving the same resolution when they print in arc motions.^[^ ^28^ ^]^ The calculated % geometrical accuracy ranges from 95.2 ± 1.1% to 103.8 ± 1.0% and the height accuracy is 95.6 ± 1.7%. These results collectively demonstrate that the printed structures possess high shape fidelity, supporting the applicability of our formulation and printing conditions for reliable fabrication.

### 3.8 Water‐Actuated Shape Transformation of the Patches

The 4D printed patch should be small in size to fit into a capsule to enable oral administration, yet be able to be actuated by water in the stomach post ingestion. The ability of agarose to deform upon dehydration and subsequently regain its shape upon rehydration^[^ ^13^ ^]^ is a key feature of the hydrogel patch. This property is leveraged to create a patch that can be compactly deformed for easy ingestion and minimally invasive delivery to the stomach. Once inside the gastric environment, where it encounters the high moisture content and specific pH conditions, the patch should rehydrate, expand and recover its shape, conforming closely to the gastric mucosa and ensuring effective contact with the target tissue.

#### 3.8.1 Effect of the Drying Conditions on the Shape Transformation

**Figure 7:**
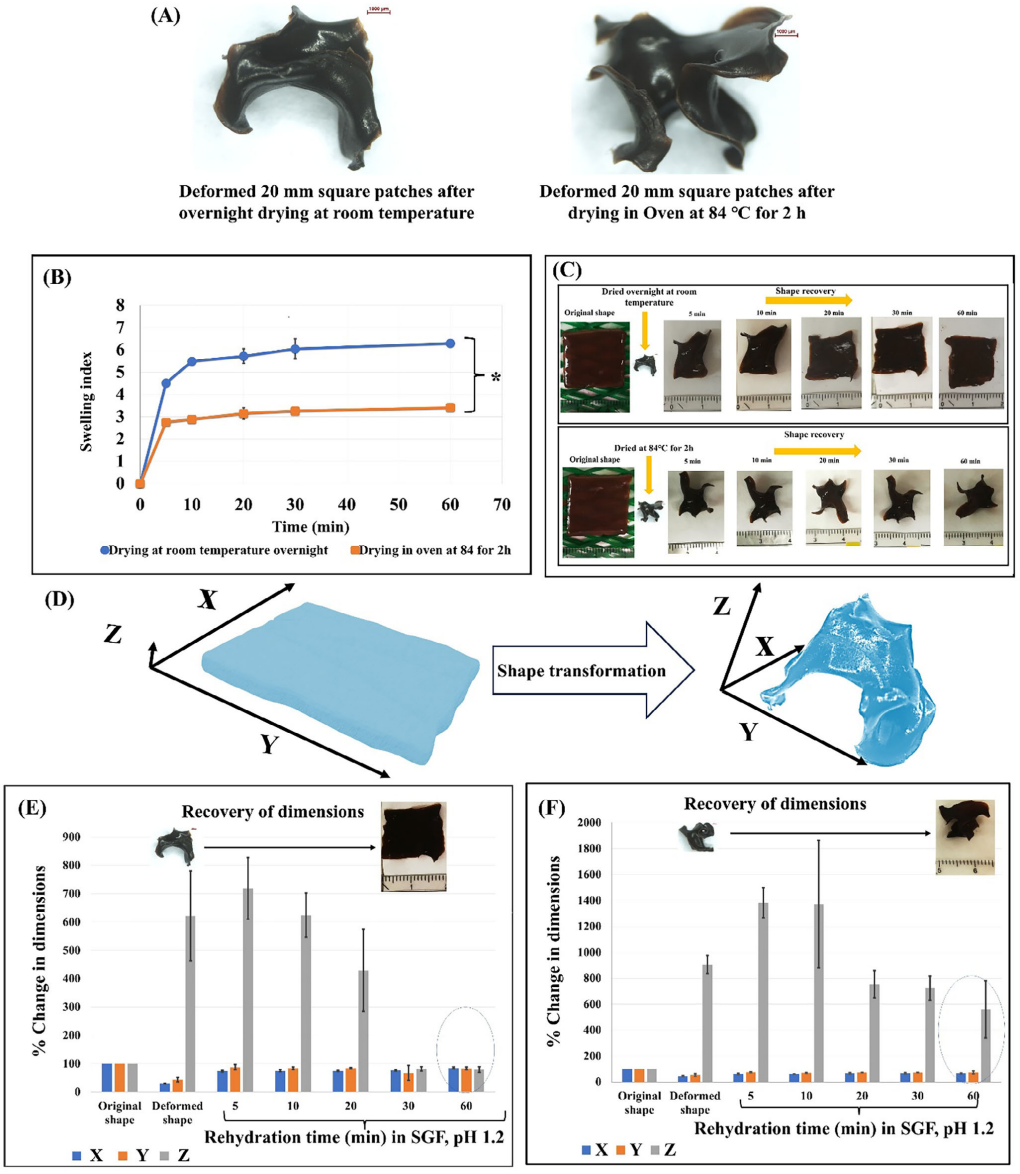
A) The 3D printed 20 mm square patches deformed into temporary structures upon drying at room temperature overnight (left) or in an oven at 84 °C for 2 h (right), scale bar = 1000 µm. B) Swelling indices of the deformed patches after rehydration in SGF, (*) indicates statistically significant differences by Student's *t*‐test. C) Photographic illustration of the rehydration of the patches (dried at room temperature overnight or in an oven at 84 °C for 2 h) in SGF. D) Schematic illustration of the 3 recorded axes of the patch. The % change in dimension upon drying/hydration of the patches E) dried at room temperature overnight and F) dried in an oven at 84 °C for 2 h. Results are expressed as means (n = 3) ± SD.

We found that the shape recovery behavior of AG/CH/SP hydrogels is highly dependent on the drying conditions. We tested the dehydration of the printed, 20 mm patches in two different conditions, either slowly at room temperature overnight or rapidly in an oven (84 °C for 2 h). Both conditions were capable of yielding small deformed structures (**Figure** **7**A), however, drying at room temperature produced smaller compacted structures in a dimensions of 6.0 ± 0.3 × 8.6 ± 1.5 × 4.2 ± 1.1 mm compared to the rapid drying process in the oven which produced a bigger structures with dimensions of 9.3 ± 1.3 × 10.7 ± 0.2 × 6.2 ± 0.5 mm. The shape transformation of the composite hydrogel patches involves complex shape change behavior which is better described by combined shrinking and curling, according to the taxonomy of shape changing materials reported by *Nam* et al.,^[^ ^64^ ^]^ which eventually lead to formation of compact deformed structures (Figure S5, Supporting Information). As shown in Figure 7A and Figure S5 (Supporting Information), the shape transformation results from initial concave deformation (curling) upon drying due to differential water evaporation across the patch layers. Water evaporates faster from the upper layers than from the bottom layers on the grid. This step is followed by less predictable shape changes that finally result in random individual shapes. Nevertheless, all the patches reproducibly shrank to compact geometries and thus fulfilled a key design principle for our intended pharmaceutical application (e.g., capsule or minimally invasive delivery). Thus, we judged the reproducibility of the deformation in terms of upward curling and shrinkage to specific compact form with dimensions that fall well within the capacity of a standard capsule size 0. Upon rehydration of the dried patches in SGF, it is obvious (Figure 7B) that there is a statistically significant difference in the swelling profile between both groups, as drying the patches at room temperature allows for more rapid water uptake, when rehydrated, than drying in an oven (*p* < 0.05). In order to assess the deployment ability of the patches, the 3 axes (as shown in Figure 7D) were recorded at different time points and expressed as the % change relative to the dimension of the original shape of the patch (Figure 7E,F). Patches dried at room temperature deployed and regained their original shape when immersed in SGF within 30 min compared to the oven dried patches that failed to deploy and had not regained the original printed structure after 60 min. This signifies that the faster drying at high temperature made the polymer chains more rigid hindering their water actuation capacity. This may be attributed to the removal of both free and hydrogen‐bonded water which may destroy the reversible switchable domains necessary for shape recovery. DSC results gave us insight into this behavior as it showed that, directly after water evaporation, an exothermic crystallization or structural reorganization process occurred. This can justify the rigidification of the polymeric chain and the failure to achieve shape recovery if harsh drying conditions are used. Hence, drying the 3D printed patches at room temperature was set as the optimum drying condition for the preparation of the deployable stomach patches.

**Figure 8:**
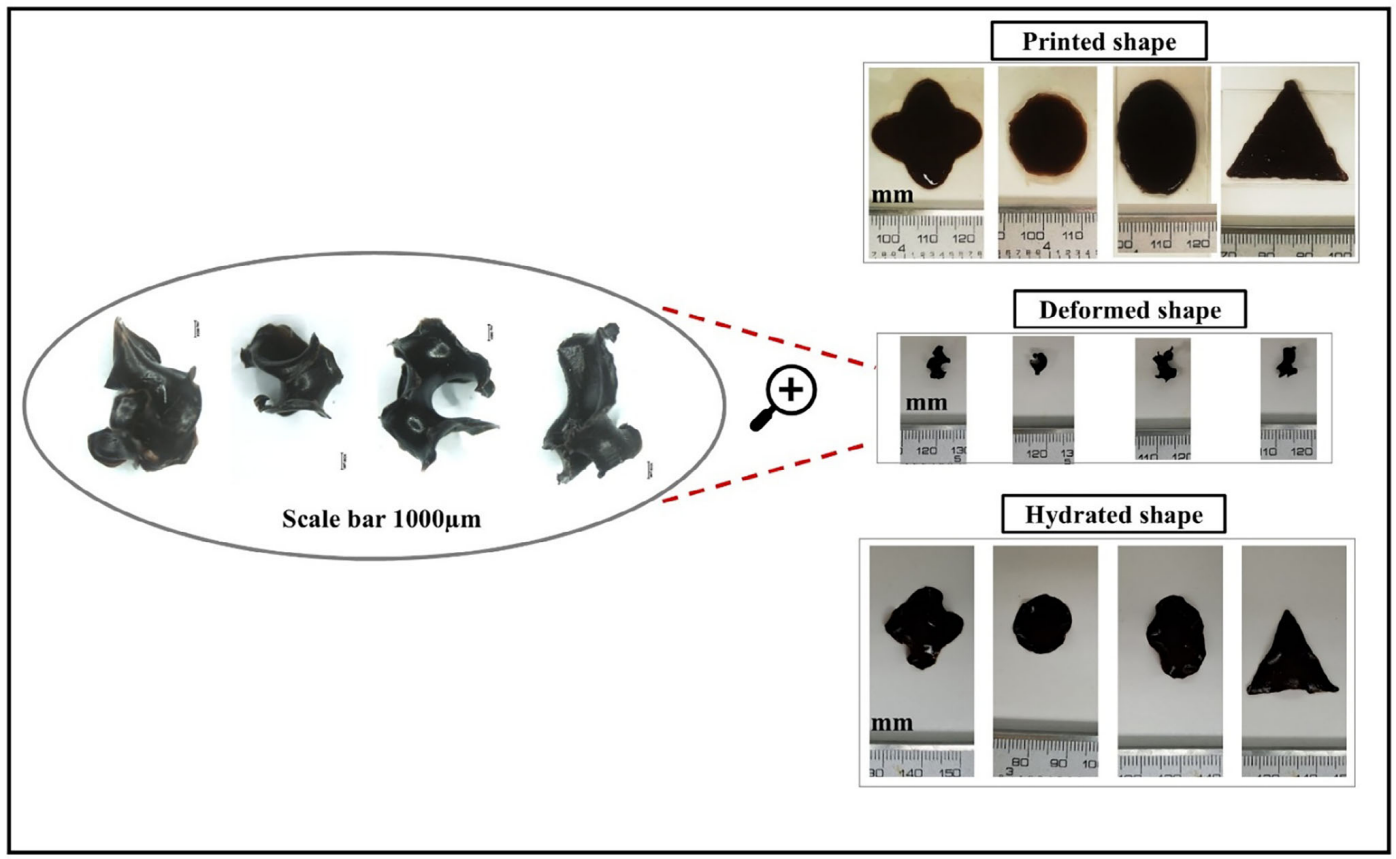
3D printed, 3‐layered patches of different shapes and sizes, deformed into a temporary structures upon drying at room temperature overnight and their respective rehydrated form after 1 h in SGF.

**Figure** **8** shows the feasibility of 3D printing to design patches of different shapes and different size with curling‐shrinking/deployment capabilities that could be printed on demand. The observed the shape transformation is driven mainly by the intrinsic properties of agarose, which alters its mechanical and structural characteristics in response to changes in hydration levels. The ability to design materials that can undergo such shape transformations is a significant advantage for biomedical applications, as it allows for the creation of devices that can be easily transported to the target site and then activated by the body's own environment.

### 3.9 Effect of Rehydration Medium on the Swelling and Shape Transformation

**Figure 9:**
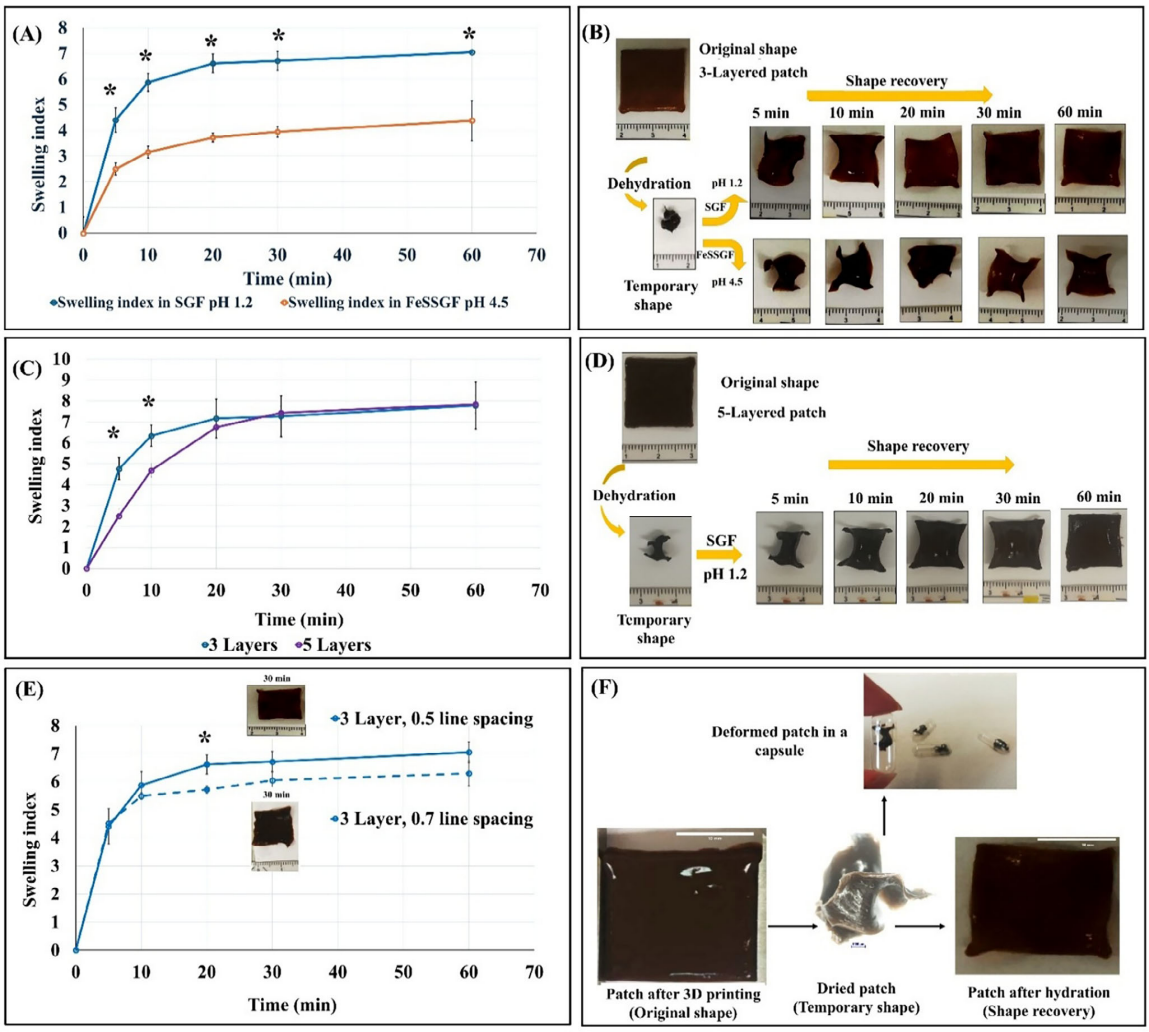
The swelling profile of the 3D printed patches showing: A) the effect of rehydration medium. B) Photographic illustration of the rehydrated 3‐layered patches in SGF and FeSSGF with time. C) The effect of the number of layers on the swelling index of the patches in SGF. Results are expressed as means (n = 3) ± SD. D) Photographic pictures exhibits the swelling and shape transformation of 3D printed 5‐layered patch in SGF. E) The swelling profile of 3‐layered patches with 0.5 mm or 0.7 mm line spacing. F) Photographic pictures showed the shape transformation of a 3D printed patch and capsule filling. (*) indicates statistically significant differences determined by t‐test, except for time points 10 and 20 minutes in C), where the non‐parametric Mann–Whitney U test was used.

Swelling of the patches was tested at pH 1.2 or 4.5 mimicking the pH of the fasted (SGF) or the fed state (FeSSGF) (**Figure** **9**A). Swelling indices of AG/CH/SP patches rehydrated in SGF pH 1.2 were statistically significant higher than after rehydration in FeSSGF pH 4.5 (*p* < 0.05). This may be attributed to the chitosan content of the hydrogel that becomes fully protonated at pH 1.2 creating repulsive forces between the cationic polymer chains.^[^ ^65^ ^]^ Moreover, the rehydration medium affects not only the swelling profile of the patches but also the shape recovery process. As illustrated in Figure 8B, the patches rehydrated in SGF could deploy and regain the original printed shape starting from 20 min while those hydrated in FeSSGF remained partially deformed even after 60 min.

### 3.10 Effect of Layers Number or Line Spacing on the Swelling and Shape Transformation

The SGF swelling profiles of AG/CH/SP patches printed with either 3 layers or 5 layers are displayed in Figure 9C. Patches consisting of 3 layers swelled faster and showed statistically significant higher swelling indices than the 5‐layered ones (5 and 10 min, *p* < 0.05). This is justified by the slower diffusion of the medium through the thicker patches. Nevertheless, both patch designs showed comparable swelling profiles starting from 20 min. Changing line spacing in a 3 layered patches (0.7 or 0.5 mm) did not cause significant effect on the swelling profiles of the patch except at 20 min (Figure 8E).

### 3.11 Effect of Enteric Coating on the Swelling and Shape Transformation

**Figure 10:**
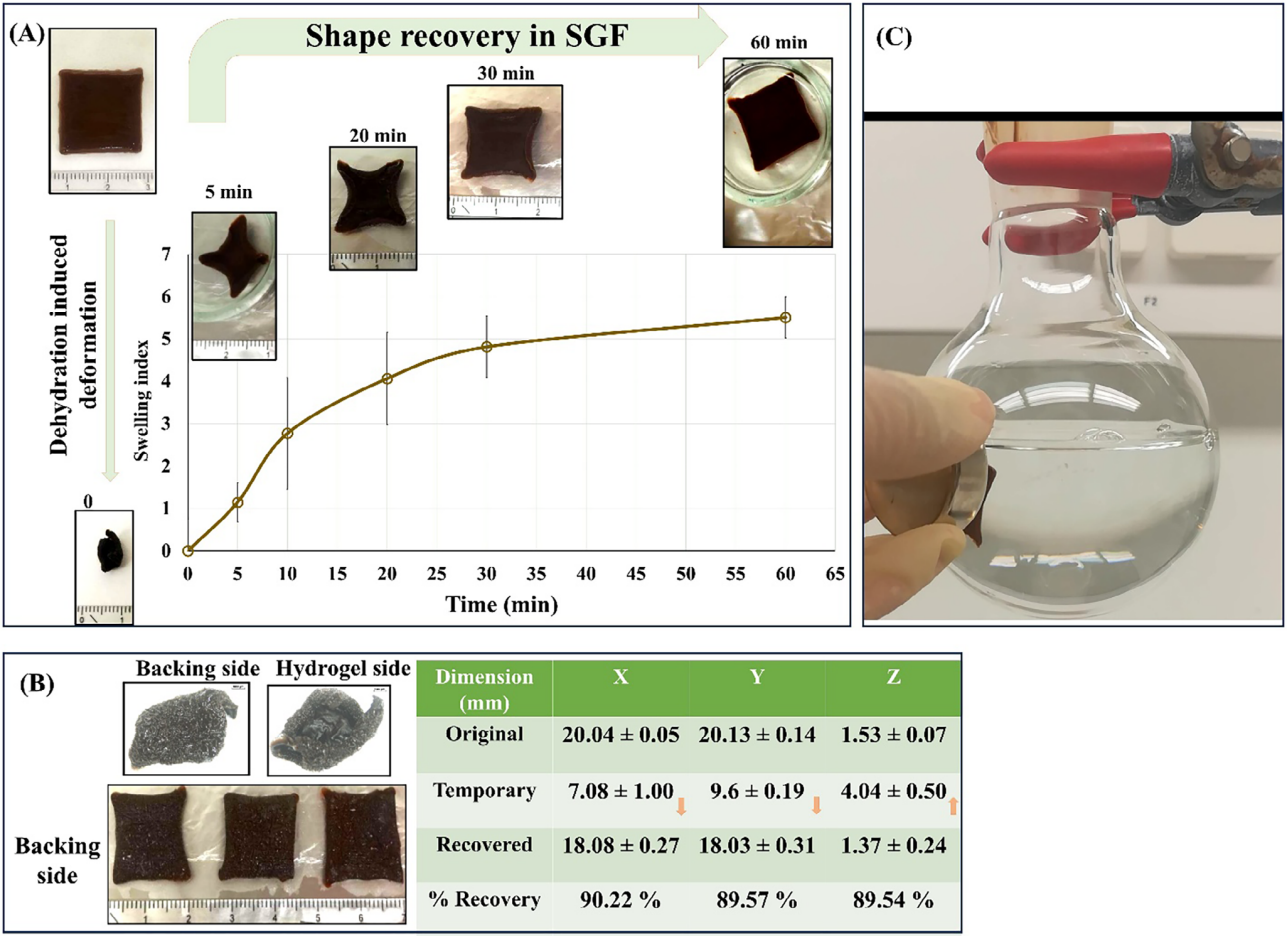
A) The swelling profile and shape recovery of the deformed 3D printed patches after enteric coating of one side of the patch. B) Photographs of the deformed and deployed patches with the change in dimensions (mm) upon dehydration/hydration in SGF, respectively. The backing layer exhibiting the flexibility of Eudragit FL layer in both the deformed and deployed patches. Results are expressed as means (n = 3) ± SD. C) Photographic image shows the possibility to navigate the swollen patch in SGF with a magnet.

The selection of the polymer to be used as a backing layer for the 3D printed patch was a very critical step in designing our deployable hydrogel patches. That polymer should act as an enteric coat, adhere strictly to the hydrogel without separation and should exhibit excellent flexibility to deform, deploy and adapt to the shape conformation of the 3D printed patches. These exceptional features were found in the novel Eudragit FL 30 D‐55 (Ethyl acrylate and methyl methacrylate copolymer, methacrylic acid‐ ethyl acrylate copolymer, 1:1). **Figure** **10** displays the hydration actuated shape transformation of the one sided‐enteric coated patches. It is noticeable that the patches were amenable to deform into small structures and recover to the originally printed shape with Eudragit FL coat which flexibly adapted to the shape change process.

### 3.12 Drug Loaded Patches: Preparation and Physicochemical Characterization of *p*‐CoA Loaded Hydrogel

*p*‐CoA was used in this study as a model drug. In a first step, we investigated the influence of the drug on the rheology as a key property for 3D printing. The hydrogel ink containing 1% *p*‐CoA switched from sol to gel upon cooling from 80 to 20 °C showing similar thermoresponsive behavior as the pure AG/CH/SP hydrogel (Figure S6A, Supporting Information). Furthermore, the formed gel did not liquefy upon reheating from 20 to 60 °C (Figure S6B, Supporting Information). However, both the sol‐gel temperature and the storage modulus decreased significantly upon drug incorporation when compared to AG/CH/SP hydrogel (Figure S6C,D, Supporting Information). Nevertheless, *p*‐CoA loaded AG/CH/SP hydrogel showed a sufficiently high storage modulus (39.87 kPa) to enable extrusion 3D printing.^[^ ^38^, ^66^ ^]^

The DSC thermograms are shown in Figure S7 (Supporting Information). It is noticeable that pure *p*‐CoA exhibits a sharp endothermic peak at 219 °C corresponding to its melting point.^[^ ^67^ ^]^ Further, the thermogram of *p*‐CoA/AG/CH/SP gel does not exhibit this melting endotherm at 219 °C suggesting the conversion of the drug from the crystalline to amorphous form in the hydrogel matrix.

The results of FTIR analysis of *p*‐CoA and *p*‐CoA loaded gel are depicted in Figure (S8, Supporting Information). The Spectrum of *p*‐CoA exhibits characteristic bands at 3552 cm^−1^ corresponding to the stretching vibration of aromatic ‐OH and stretching vibration of CH at 2786 cm^−1^. The stretching vibration of carboxylic C═O is observed at 1725 cm^−1^ as well as out‐of‐plane and in‐plane deformation of C═O at 532 and 703 cm^−1^, respectively. Additionally, the stretching vibration of alkene C═C and the aromatic C═C are at 1638 and 1525 cm^−1^, respectively. The bands at 1430 and 1393 cm^−1^ are assigned to alkene ═C─H while the aromatic ═C─H band is at 1226 cm^−1^. Further, the peaks of the substituted benzene rings are observable at 930 and 847 cm^−1^. These results are in accordance with previous reports.^[^ ^67^, ^68^ ^]^ The characteristic bands of *p*‐CoA can be observed in *p*‐CoA/AG/CH/SP gel signifying the compatibility of the drug with the gel components. The ─OH stretching vibration band has broadened and shifted to 3260 cm^−1^ suggesting hydrogen bonding formation. The characteristic Fe─O vibration band of SPIONs can be seen at 533 cm^−1^ suggesting that incorporation of *p*‐CoA to the hydrogels did not affect Fe─O bond in SPIONs loaded hydrogel.

Similar to AG/CH/SP gel, the SEM images of dried samples of *p*‐CoA loaded AG/CH/SP gel (Figure S8, Supporting Information) revealed homogenous distribution in the gel matrix and their retention after incubating the gel with SGF for 72 h.

### 3.13 Drug Content

The drug content of the patches was 103.9 ± 7.9% of the theoretical % *p*‐CoA amount, with no individual unit outside the range of 85–115% of the average content, indicating optimum drug uniformity according to European pharmacopeia acceptance criteria.^[^ ^69^ ^]^ This confirm that the drug was properly and homogenously distributed in the gel matrix.

### 3.14 In Vitro Drug Release and Functionality of the Enteric Coat

**Figure 11:**
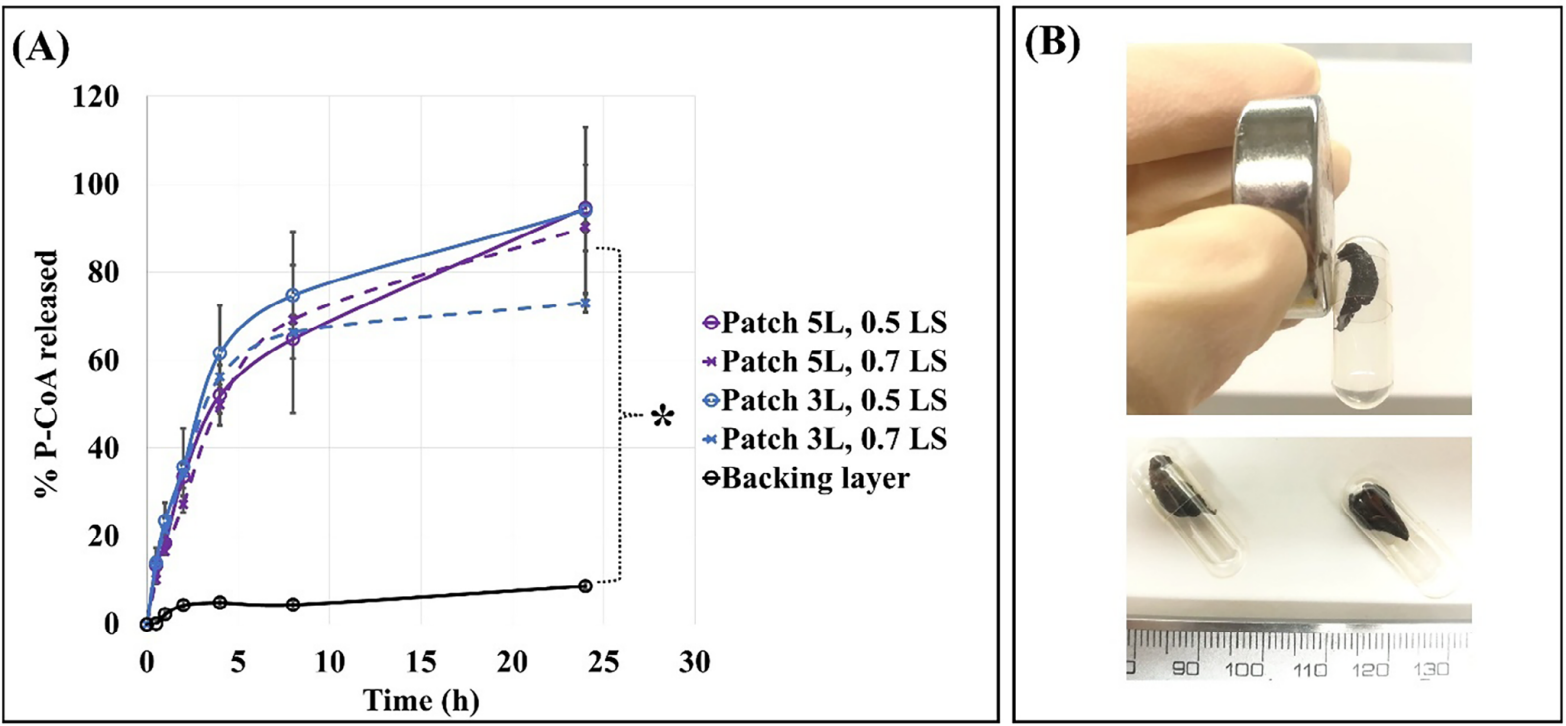
A) Release profiles of *p*‐CoA from 4 tested designed patches (L: layer number, LS: line spacing in mm). Results are expressed as means (n = 3–4) ± SD. (*) indicates statistically significant differences by one‐way ANOVA with Tukey post hoc test. B) Pictures exhibiting the deformed *p*‐CoA patches encapsulated in hard gelatin capsules and the capsule is attracted to neodymium nickel plated magnet. (scale bar in mm).

Drug release from the 3D printed patches was tested for patches with 4 printed designs that differed by the number of printed layers (3 or 5) and the line spacing (0.7 or 0.5 mm). All investigated patches had Eudragit FL coating on one side in order to generate a unidirectional drug release from the patches. The test was conducted by placing the dried deformed patches in the 60 ml SGF on a shaker at 50 rpm and 37 °C. Although this drug release set up did not mimic the intended application where the patch is planned to be placed in a close proximity to tumor, we were interested in differences relying on the patch design (**Figure** **11**). All patches released the drug over a period of 24 h with a cumulative % *p*‐CoA release ranging from 10.2 to 14.5% after 0.5 h and from 73.1 to 94.6% after 24 h. No statistically significant differences were found for the investigated 4 designs (except between 5L/0.7LS and 3 L/0.7LS at 10 min) indicating that the changes in layer number (L) from 3 to 5 or line spacing (LS) from 0.5 to 0.7 mm were not sufficient to produce dramatic changes in the release profile of *p*‐CoA. Interestingly, the designed 4 group of patches composed of different drug amount in each group which means that patches with various dosage, yet with similar release behavior, can be designed tailored to each patient's need. The patches of 5L/0.5LS, 5L/0.7LS, 3L/0.5LS and 3L/0.7LS had average total weights of 647.8 ± 22.1, 585.2 ± 13.6, 387.6 ± 26.7 and 328.7 ± 20.8 mg, respectively. The release profile of *p*‐CoA from the Eudragit FL coated side is statistically significant lower than the release from the hydrogel side for all patterns (as depicted in Figure 11). This reveals that Eudragit FL backing could be successfully utilized to limit *p*‐CoA release if applied to the surface of the patch with a maximum cumulative % drug release of 8.68 ±1.93% after 24 h.

### 3.15 Ex vivo Mucoadhesion

**Figure 12:**
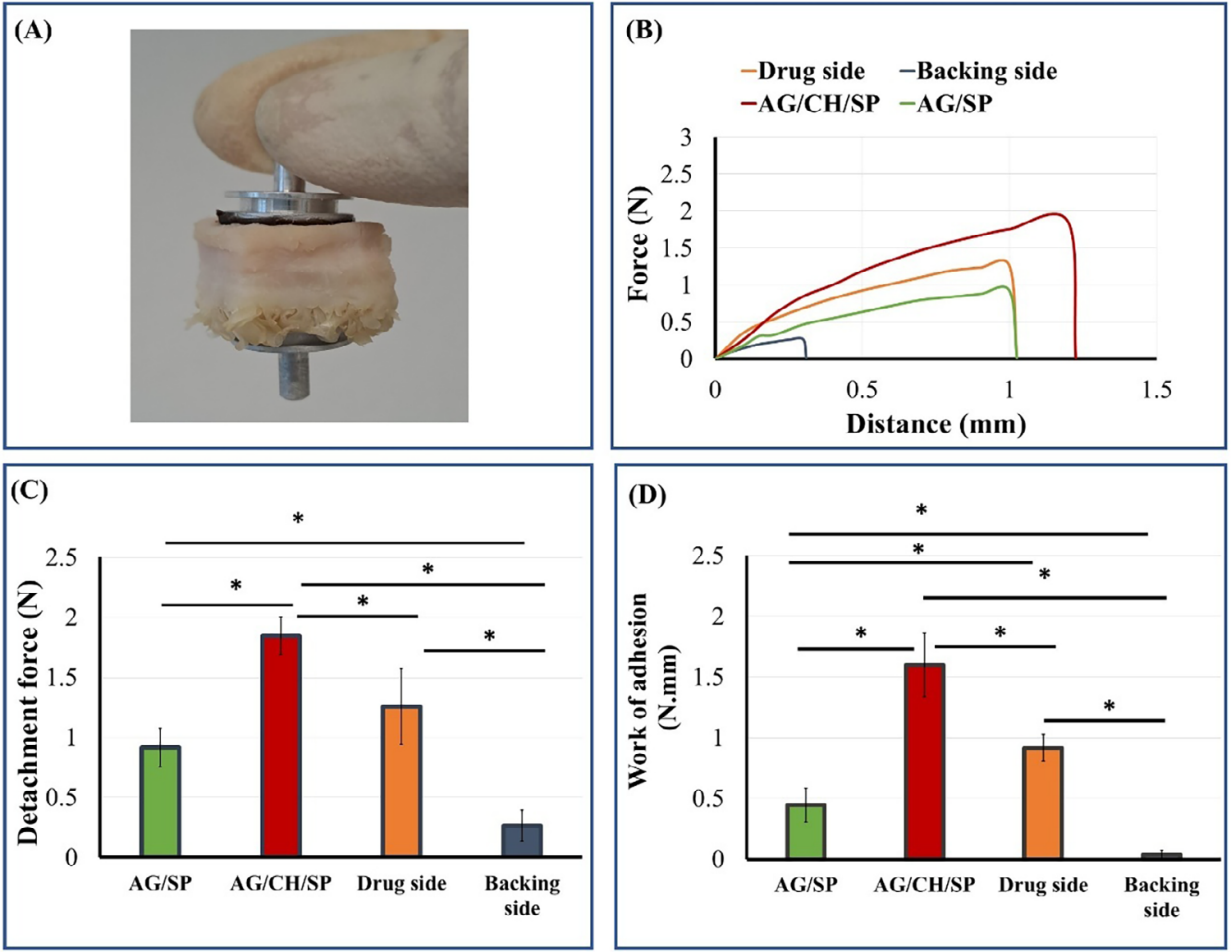
Mucoadhesion of the patches to excised cow stomach tissue: A) Photograph shows the metal holders to which the patches and stomach tissue were attached, the stomach tissue is held in this position through the mucoadhesion properties of the hydrogel side of the patch. Results of B) Force vs distance profiles, C) peak detachment force, and D) work of adhesion of mucosal side of cow's stomach tissue to either drug side (*p*‐CoA/AGCH/SP), AG/CH/SP, AG/SP, backing layer side of the patches. Results are expressed as means (n = 3–6) ± SD. (*) indicates statistically significant differences by one‐way ANOVA with Tukey post hoc test.

In order to evaluate the mucoadhesive strength of the patches we investigated adhesion strength to the mucosal side of cow stomach tissue by the hydrogel side of *p*‐CoA loaded AG/CH/SP, AG/CH/SP and AG/SP patches. Additionally, we tested the adhesion force of the backing layer side (Eudragit FL side) of the patches to the mucosa. The maximum peak force and work of adhesion was obtained in case of AG/CH/SP hydrogel side with values of 1.84 ± 0.16 N and 1.6 ± 0.26 N.mm (**Figure** **12**). Results revealed that addition of chitosan to AG/SP hydrogel significantly increased both the peak detachment force and work of adhesion of the patches (*p* < 0.01). Furthermore, it is noticeable that the peak detachment force and the work of adhesion of the backing layer side is significantly lower than of the hydrogel side in either drug loaded or plain AG/CH/SP patches (*p* < 0.01). Incorporation of *p*‐CoA acid to AG/CH/SP significantly decrease the detachment force and the work of adhesion of the patches. This may be justified by the electrostatic interaction of carboxylic group of the drug with the positively charged amino groups of chitosan decreasing their availability for interaction with mucin in the mucosal side of the stomach.^[^ ^70^ ^]^ Nevertheless, the mucoadhesion of the drug side is still significantly higher than of the backing layer side of the patch (*p* < 0.01).

### 3.16 Assessment of Tensile Strength of the Patches

**Figure 13:**
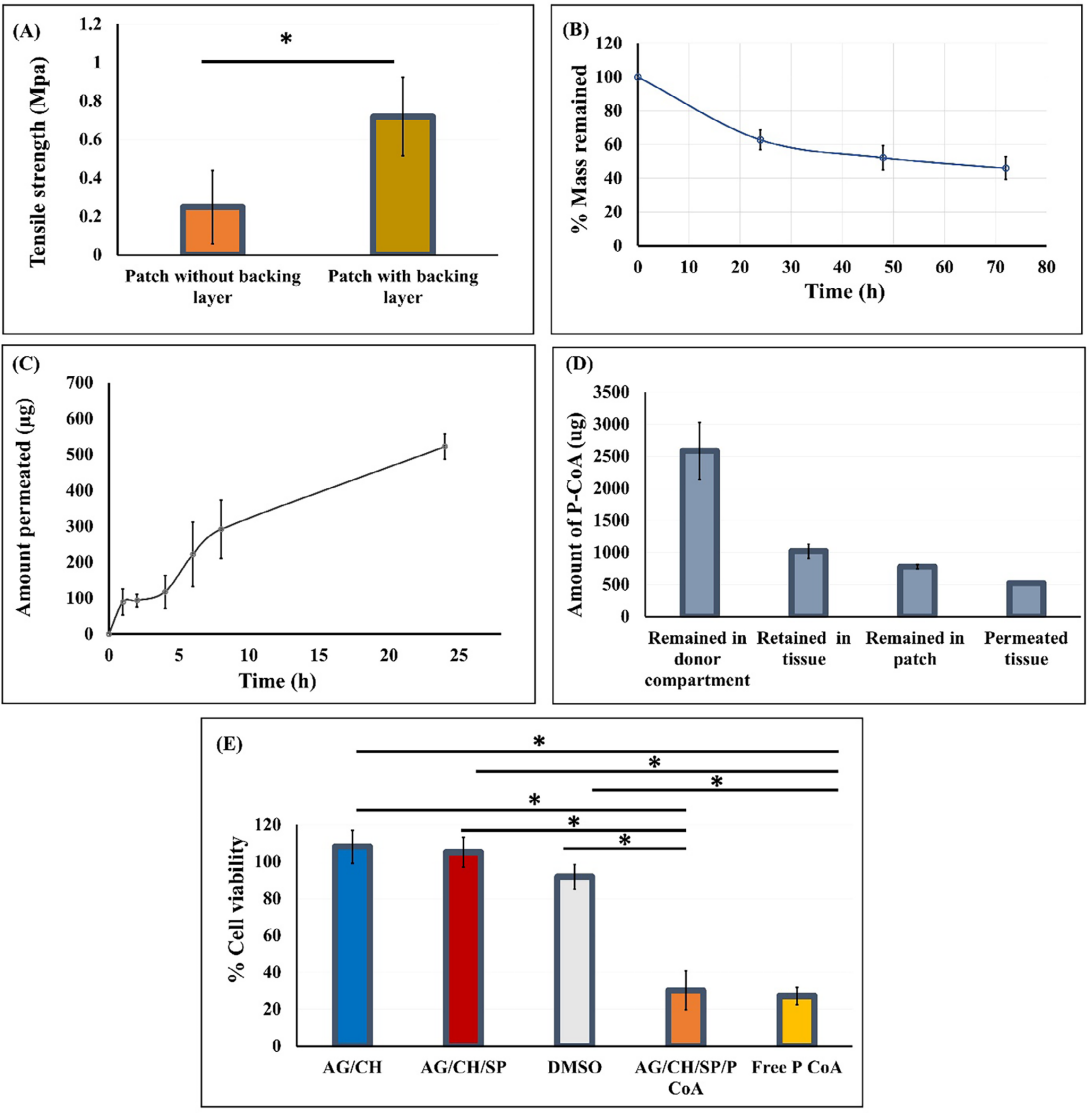
Characterization of *p*‐CoA loaded AG/CH/SP patch: A) Tensile strength of *p*‐CoA loaded patch with or without backing layer (n = 3–4 ± SD). (*) indicates statistically significant differences by Student's *t*‐ test. B) Degradation of the patch in SGF pH 1.2. C–E): Ex vivo permeation study exhibiting (n = 3 ± SD) C) permeation profile of *p*‐CoA across cow stomach tissue, D) amount of *p*‐CoA permeated across or retained in the stomach tissue as well as the amount remained in the patch after 24 h in SGF (n = 4 ± SD). E) % Cell viability of Caco‐2 cells after treatment with *p*‐CoA loaded and plain hydrogel patches, normalized to control untreated cells. (n = 3 ± SD). (*) indicates statistically significant differences by one‐way ANOVA with Tukey post hoc test.

When designing water responsive hydrogel materials, the decrease in mechanical strength post swelling or expansion needs to be considered.^[^ ^15^ ^]^ Hence, we tested the ultimate tensile strength of the 3D printed patches, with or without Eudragit FL backing layer, after swelling for 6 h in SGF and the result is shown in **Figure** **13**A. Both patches exhibit high toughness with tensile strength of 0.25 ± 0.19 and 0.72 ± 0.21 MPa for the patches without or with backing layer, respectively. Obviously, Eudragit FL coating of the patches significantly increased the tensile strength values (*p* < 0.05). Thanks to the excellent adhesion properties of the Eudragit FL, it tightly adhered to the hydrogel imparting a high mechanical strength to the patches.

### 3.17 Degradation of the Patches

The degradation of *p*‐CoA loaded AG/CH/SP patch was studied for 96 h in SGF and the resulting degradation profile of the patches is presented in Figure 13B. This test was conducted to check the fate of the patches after the drug is released. After 72 h incubation in SGF, 46.00 ± 6.82% of the patch mass remained. The test was stopped at 96 h as parts of the hydrogel layers of the patches were found to be broken into pieces. Though agarose exhibits poor degradation rate,^[^ ^71^, ^72^ ^]^ the incorporation of chitosan, SPIONs and *p*‐CoA to agarose matrix might be the reason for this accelerated degradation as they changed agarose hydrogel matrix. Moreover, the diffusion of drug and loosely bound polymeric chains out of the matrix with time also contributes to the degradation. Nevertheless, SPIONs were retained throughout the testing period, as verified visually by the consistent color, absence of particulate leaching in the medium and previously concluded from SEM images (Figure 5). This degradation pattern of the patches is favorable to facilitate their safe evacuation from the stomach after drug release.

### 3.18 Ex vivo Drug Permeability

The permeation of *p*‐CoA across excised cow stomach was studied in an open‐membrane model.^7^ In this Ex vivo study, the patches were placed in the donor compartment with their drug releasing side facing the mucosal tissue. The patches were not manually adhered to the mucus but were instead allowed to gradually uncurl and expand in contact with the tissue simulating the post‐ingestion case where the patch reaches the stomach in the deformed state. Further, this approach was chosen to simulate the scenario where the patch is delivered initially close to the tumor site with magnetic guidance. This was also to minimize the variability introduced by placement of the patch by manual force. Figure 13C depicts the permeation profile of the released *p*‐CoA from the patches with time. There is a gradual increase of *p*‐CoA amount permeated across stomach tissue with time. The cumulative amount *p*‐CoA that permeated through the stomach tissue ranged from 89.4 ± 35.8 µg (1.8%) after 1 h to 535.7 ± 39.3 µg (10.6%) after 24 h. Large quantities of the drug (Figure 13D), however, were deposited in the stomach tissue (1023.1 ± 443.8 µg) after 24 h. This means that ≈ 21% of the drug were available within the tissue to exert a local anticancer effect. This is favorable as the primary reason for gastric cancer treatment failure, is the difficulty to deliver sufficiently high drug amounts to the target tissue.^[^ ^73^ ^]^ The larger drug fraction was retained in the donor compartment which is in accordance with the observation Bandi and Venuganti who reported on testing the permeation of oxaliplatin from their developed local gastric patch.^[^ ^7^ ^]^ Overall, the results revealed that *p*‐CoA could be gradually and continuously released from the patches, deposited within it with a small amount that permeated across the tissue (only 10.6%) which would minimize systemic absorption and allow for more localized effects. Nevertheless, if the patches were adhered to the tissue, this would have limited the escape of the drug to the donor compartment. This is believed to be better in the real case scenario as the magnetization gripping force can permit the patch to adhere to the mucus after the deployment of the patch.

### 3.19 Evaluation of the Cytotoxicity of the Patches

The cytotoxicity of the fabricated hydrogel patches was evaluated on Caco‐2 cells. The patches were prepared without the final addition of Eudragit FL coating, as the coating dissolved in the pH of cell culture medium and caused cytotoxicity providing irrelevant results which would not be the case as it is designed to be intact in acidic environment as in our intended gastric application. The inhibition of Caco‐2 cell growth post incubation with plain hydrogel patches, *p*‐CoA loaded patch as well as free *p*‐CoA is shown in Figure 13E. No cytotoxicity was found for any of the API‐free patches with cell viability of 108% ± 8.8% and 105% ± 8% for AG/CH and AG/CH/SP patches. On the other hand, the free *p*‐CoA acid or *p*‐CoA loaded AG/CH/SP patches inhibited cell growth with remaining viability of 27% ± 5% and 30% ± 11% of Caco‐2 cells, respectively with no significant differences between both treatments. This indicates that incorporation of the drug in the hydrogel patch and the printing process did not affect the cytotoxic effect of the drug.

Overall, in this work, we propose a 3D printed patch as a platform for delivering anticancer drugs to manage gastric tumors. The design of our patch complies with the 4D printing concept which permits the fabrication of stimulus responsive, shape changeable materials taking the time as the fourth dimension.^[^ ^61^ ^]^ This allows the fabricated patches to exist in relatively small configuration for easy administration either in a capsule or through minimally invasive endoscopic insertion. The deployed patch is intended to cover an early or advanced stage tumor or to be used as adjuvant therapy to eradicate the remaining cancer cells present after surgical removal of the tumor. In such cases, the patch will allow for localized treatment via the drug releasing printed matrix. The incorporated SPIONS provide the patch with the option to be magnetically guided to the target site where it can release its loaded drugs to the tumor tissue at augmented concentrations. Tailoring the size, shape, dose and composition of the patches to fit each patient´s need complying with personalized medicine is implemented via 3D printing. Since the application of 4D printing in drug delivery is still in its infancy, there are, however, a number of points that should be considered for future development. For instance, it would be advantageous to accelerate the shape recovery time and the placement of the patch to prevent premature evacuation from the stomach before deployment. Nevertheless, the magnetic properties of our patch are believed to act as another gastroretention strategy along with mucoadhesive properties to retain the patch in the desired region till the deployment is completed and the patch can be positioned adequately. The deformation of patches follows a predictable pattern driven by differential water loss, particularly when the samples are dried on a mesh support, leading to upward curling. This upward curling as well as compact geometries are sufficient for our intended application, which requires consistent reduction in patch dimensions that meet the size requirement for oral capsule delivery and that these patches recover their original shape upon rehydration in SGF within a reasonable timeframe. Future studies may therefore focus on generating suitable computational models for dimensional prediction of temporary shapes, especially, if huge and various possibilities for shape and size of patches are offered and can be generated by 3D printing.

The mucoadhesion of the patch needs to be tested in vivo to obtain more reliable data verifying the possible localization of the patches close to the tumor site in the presence of housekeeping waves which are the strong contractions propagating in the distal part of the stomach.^[^ ^74^ ^]^ Nevertheless, the magnetic properties of our patch are believed to act as another gastroretention strategy along with mucoadhesive properties to retain the patch in the desired region till the deployment is completed and the patch can be positioned adequately. Other mucoadhesive materials may be tested in the future studies, for instance, modification of chitosan to chitosan‐hydrocaffeic acid conjugate has recently been shown to retain mucoadhesive to mice gastric mucosa for 8 days in vivo.^[^ ^9^ ^]^ Sustaining the drug release to longer periods is another aspect of future improvement. One advantage of our design concept is the versatility of 3D printing that allows to adapt our system to the requirements of different APIs and different size. For instance incorporating additional polymers can be considered, even in the form of 3D printed small drug loaded depots that can be embedded in the hydrogel matrix during printing,^[^ ^74^ ^]^ not to hinder the shape changing properties of the platform patch. In such a case, the hydrogel layer may act as a distributive matrix for the drug which can allow large surface area of the tumor to be covered. Further, incorporation of SPIONs in our patch offers not only magnetic responsiveness for targeted delivery but also potential for non‐invasive monitoring via magnetic resonance imaging (MRI) or magnetic particle imagining (MPI). Given their established use as imaging contrast agents in clinically approved formulations (e.g., Endorem)^[^ ^75^ ^]^ and various research reports,^[^ ^76^, ^77^, ^78^ ^]^ our system holds promise for image‐guided placement and future clinical translation. This can be beneficial to ensure that the patch is placed in the right orientation where the mucoadhesive site is facing the mucus tissue depending on the shape‐morphing property of our 4D printed patch. Upon shape change, the patch assumes a predictable shape: a concave curvature on the hydrogel (adhesive) side and a convex curvature on the backing side. This distinct morphology enables visual discrimination of the patch orientation, especially under imaging or endoscopic guidance. Overall, our multi‐material patch serves as a platform for future improvement, providing a basis for further optimization and development. The combination of mucoadhesion, magnetic guidance and unidirectional drug release in a 4D printed patch to the best of our knowledge has not been reported before. Moreover, Eudragit FL coating to the hydrogel patch, with strong adhesion and flexibility, represents a notable advancement in the field of unidirectional drug delivery hydrogel systems. This approach addresses a common challenge which is the lack of sufficient bonding between the hydrogel and the hydrophobic coating resulting in separation of the coating from hydrogel layers. Hence, researchers had to use precoating solutions involving multi‐stages coating procedures.^[^ ^79^ ^]^ Nevertheless, the food intake of the patients need to be adapted in a way that keeps the pH of the stomach (<5.5) preserving Eudragit layer intactness. Finally, our proposed patch represents a potential candidate for site‐specific and personalized drug delivery to support gastric cancer management via drug release directly at the tumor site.

## 4 Conclusion

The deployable, 3D printed hydrogel patch is a combination of several cutting‐edge technologies, including 3D printing, responsive hydrogels, enteric polymers, and magnetic nanoparticles. This multifaceted approach may address the key challenges associated with the treatment of gastric cancer, including the need for targeted delivery, localized therapeutic action, and minimal invasiveness. By providing a platform for the direct and sustained release of therapeutic agents at the tumor site, the hydrogel patch has the potential to improve treatment outcomes and reduce the side effects commonly associated with conventional therapies. Furthermore, the patch's ability to be magnetically guided and positioned offers a level of precision that is critical for effective cancer treatment, particularly in the complex and dynamic environment of the stomach. In conclusion, the development of a deployable hydrogel patch for stomach cancer therapy represents a significant advancement in the field of localized drug delivery. This innovative approach has a great potential to create highly effective and targeted treatment modalities. As research in this area continues to evolve, the deployable hydrogel patch may contribute to a next step in the development of new complementary therapeutic approaches to treat stomach cancer.

## Conflict of Interest

The authors declare no conflict of interest.

## Author Contributions

Dina B.Mahmoud, PhD performed conceptualization (Lead); Formal analysis (Equal); Funding acquisition (Lead); Investigation (Lead); Project administration (Equal); Validation (Equal); Visualization (Lead); Writing–original draft, review, editing (Lead). Martin Börner, PhD: Investigation (Supporting); Methodology (Supporting); Writing – original draft (Supporting); Writing – review & editing (Supporting). Christian Wölk, PhD: Data curation (Supporting); Supervision (Supporting); Validation (Supporting); Writing – review & editing (Supporting). Michaela Schulz‐Siegmund: Data curation (Supporting); Funding acquisition (Equal); Project administration (Equal); Resources (Lead); Supervision (Lead); Validation (Equal); Writing – review & editing (Lead).

## Supporting information



Supporting Information

## Data Availability

The data that support the findings of this study are available from the corresponding author upon reasonable request.
