# Supplementary material for: A Deployable 4D Printed, Mucoadhesive and Magnetically Guided Patch for Local Therapy of Gastric Cancer
Source: Adv Healthc Mater. 2025 Jul 26;14(28):e01085. doi: 10.1002/adhm.202501085 (PMC12581888; doi:10.1002/adhm.202501085)
Supplement: Supplementary file 1 — Supporting Information [file ADHM-14-0-s001.docx]

**Supplementary data**

**
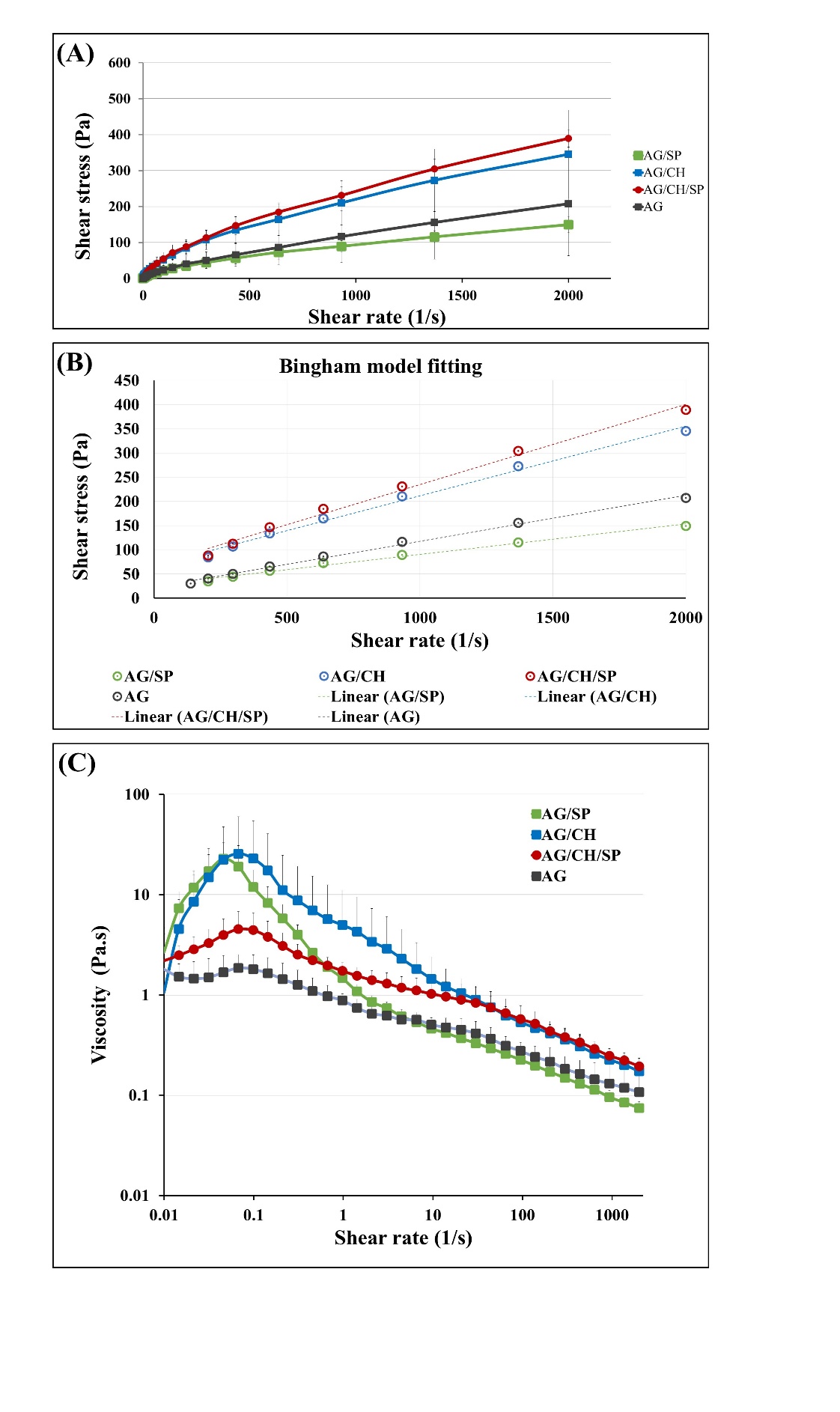
**

**Figure S1.** Rheological evaluation of the hydrogels at determined at the printing temperature (73 ℃) showing (A) Flow curves as shear stress vs shear rate (B) Bingham model fitting (C) Shear thinning behavior of the hydrogels as shown in the viscosity against shear rate plot. Curves expressed as mean values (n = 3).

**Table S1.** Parameters of Bingham model and power law model fitting of shear rate and shear stress data (n=3)

| **Hydrogel** | **Bingham yield point (τ_B_)**  **(Pa)** | **Bingham viscosity (η_B_)**  **(Pa.s)** | **Flow index (n)** |
| --- | --- | --- | --- |
| AG | 20.9 ± 7.2 * | 0.10 ± 0.08 | 0.72 ± 0.08 |
| AG/CH | 69.4 ± 2.9 | 0.14 ± 0.06 | 0.62 ± 0.20 |
| AG/SP | 19.5 ± 0.3 * | 0.08 ± 0.03 | 0.65 ± 0.02 |
| AG/CH/SP | 67.1 ± 24.1 | 0.17 ± 0.01 | - 1. 0.03 |

(*) indicates statistical significant differences from AG/CH or AG/CH/SP (p < 0.01) determined by One-way ANOVA test with post-hoc Tukey, Results expressed as mean (n = 3 ± SD).

**
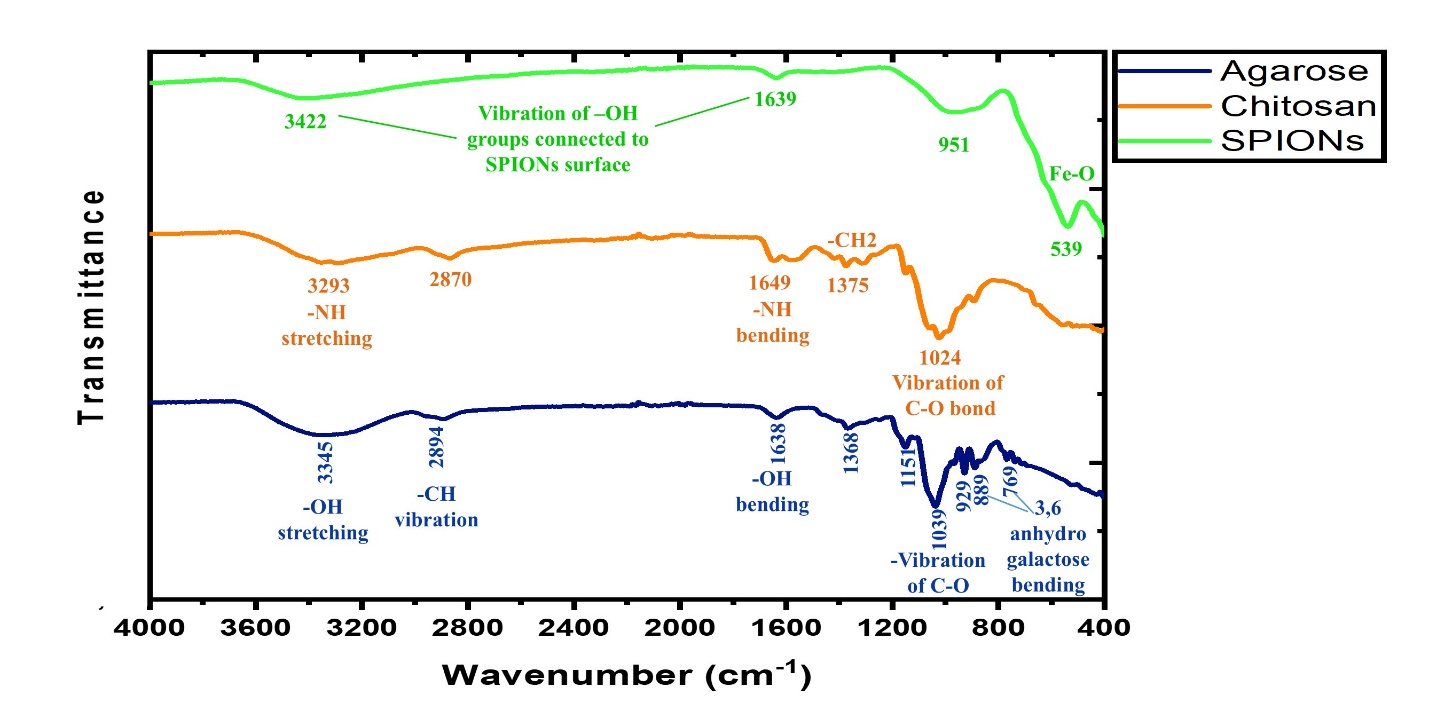
**

**Figure S2.** FTIR spectra of agarose, chitosan and SPIONs.

**
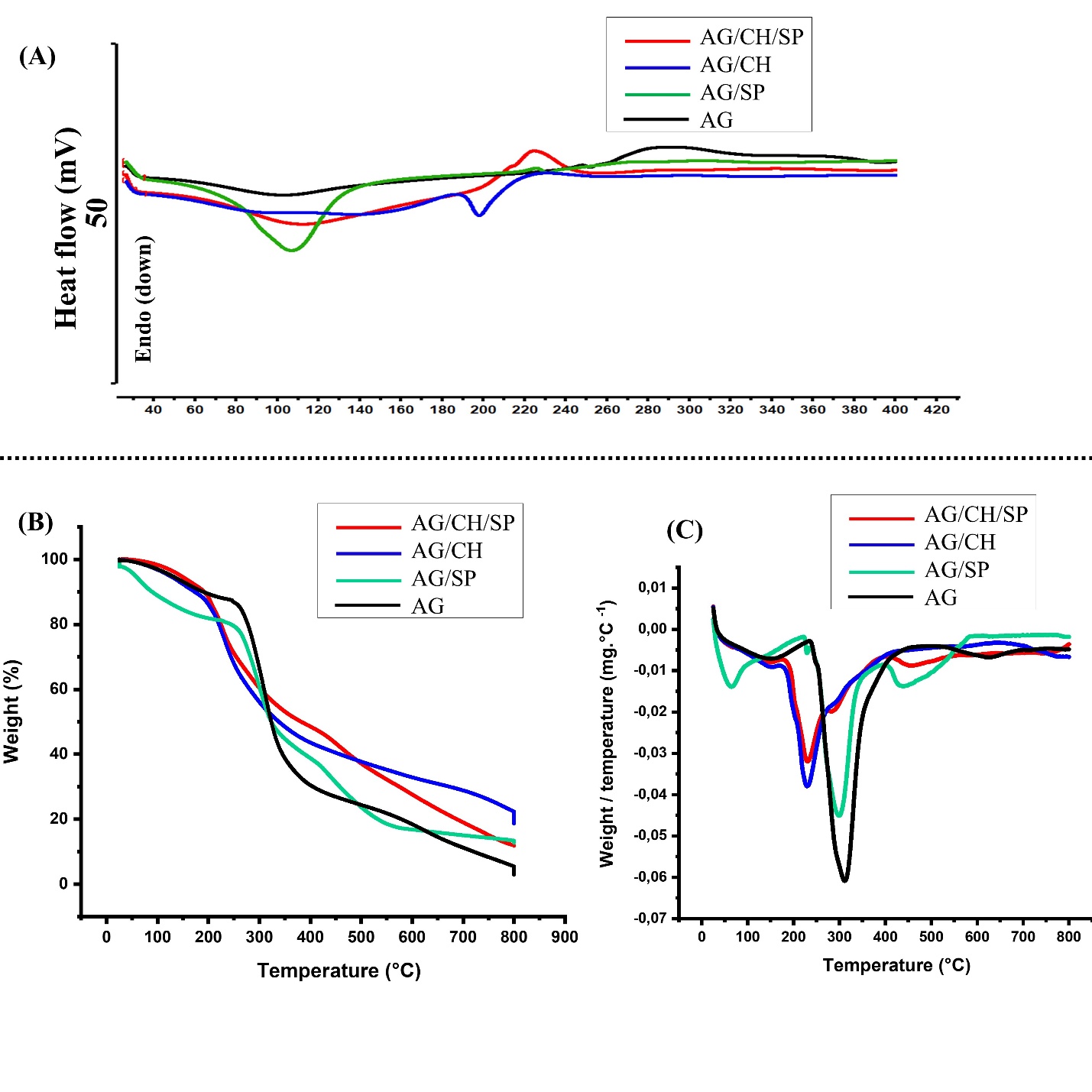
**

**Figure S3.** (A) DSC analysis of AG and the composite hydrogels AG/CH, AG/SP, AG/CH/SP. Results of TGA analysis of the prepared hydrogels AG, AG/CH, AG/SP and AG/CH/SP expressed as: (B) % Residual weight vs temperature (C) first derivative curves.


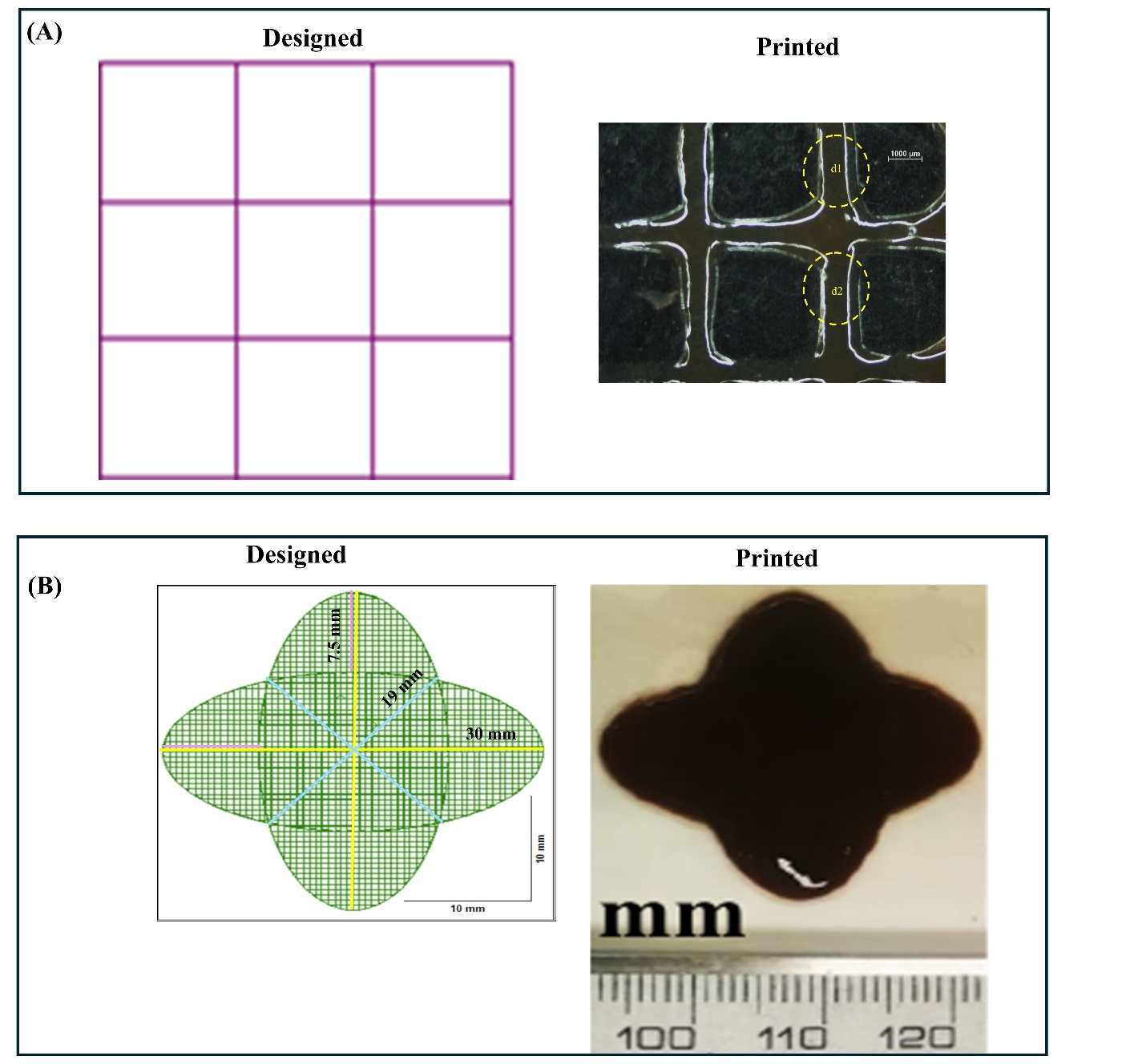


**Fig .S4.** CAD designs vs 3D printed structures (A) grid pattern showing filament width at the intersection (d1 & d2) (B) two intersecting ellipses, 4 layered structure that was used for geometrical accuracy calculations .


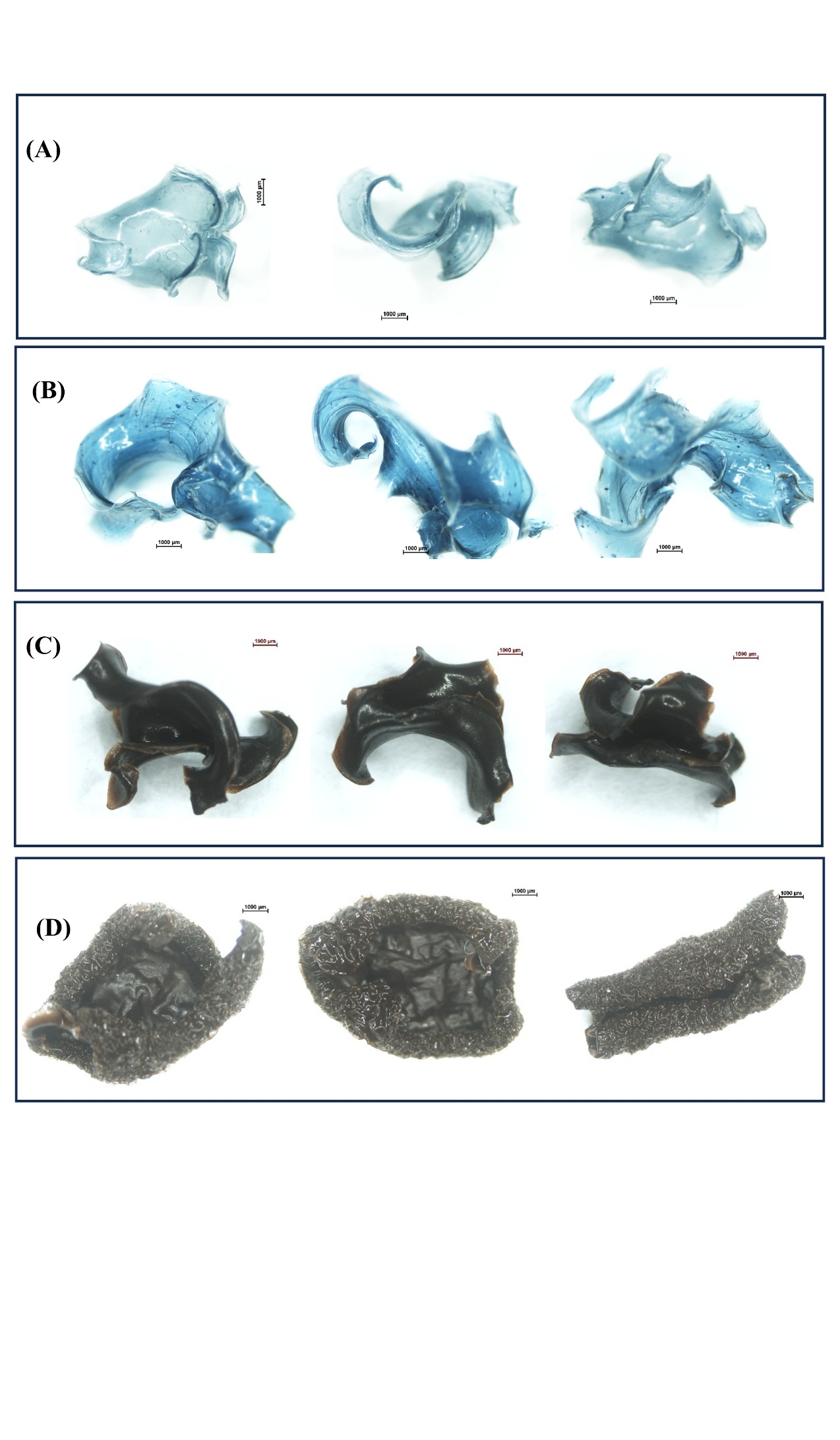


**Figure S5.** Images depict the deformation behavior for three independent samples upon drying of (A) Square shaped patches of 2 cm 3D printed of AG hydrogel (B) Triangle patches of 3 cm, 3D printed of AG/CH composite hydrogel gel (C) Square shaped patches of 2 cm 3D printed of AG/CH/SP hydrogel (D) Eudragit FL coated patches showing the hydrogel side on top; suggesting that the shape change has a relatively consistent manner in terms of upward curling pattern and volumetric shrinking to a compact form suitable for a standard size 0 capsule filling). AG and AG/CH hydrogels were loaded with a dye for better visualization. Scale bar is 1000 µm.

**
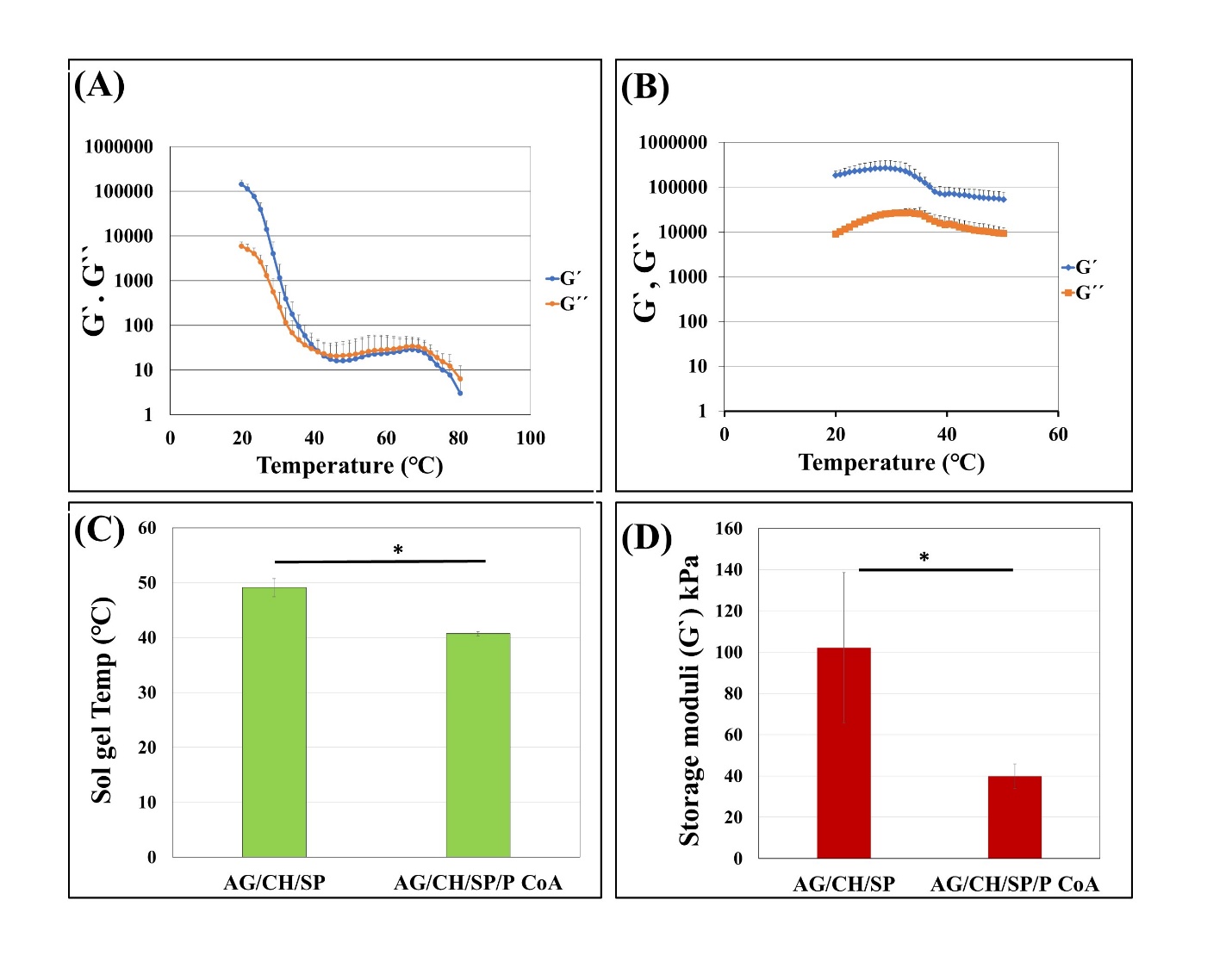
**

**Figure S6.** Rheological evaluation of AG/CH/SP and *p*-CoA loaded AG/CH/SP hydrogels shows storage (G`) and loss (G``) moduli of the hydrogels determined via temperature sweep tests (A) upon cooling from 80 to 20 °C (n = 3-4), (B) upon heating from 20 to 60 °C (n = 3-4), (C) storage moduli of the hydrogels at 25℃ (n = 3-4). Results expressed as means, error bars in (A) and (B) represent only the positive standard deviation (+SD) due to the logarithmic Y-axis for clarity. In (C), results are shown as mean ± SD. (*) indicates significance difference (p < 0.05) determined by Student's *t*- test.


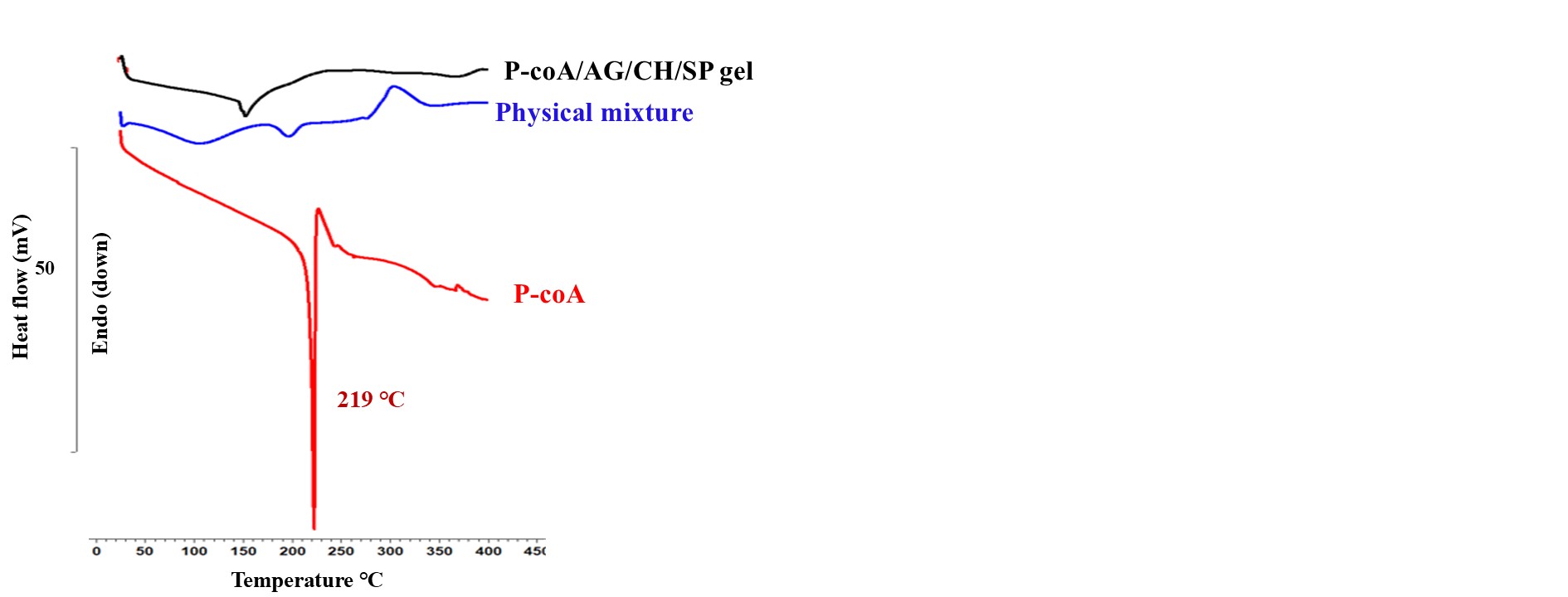


**Figure S7.** DSC thermograms of *p*-CoA, *p*-CoA loaded hydrogel and the respective physical mixture of all components.


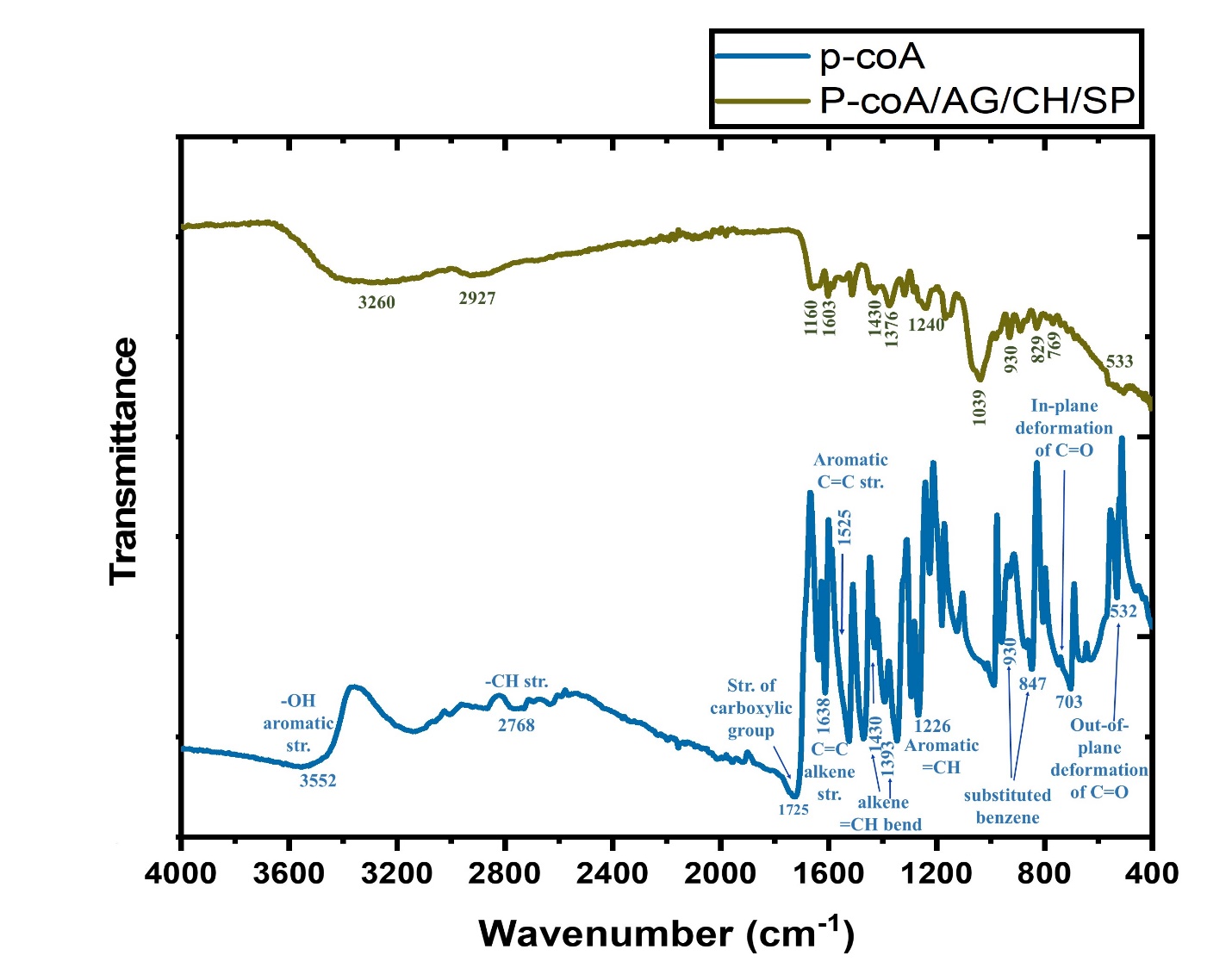


**Figure S8.** FTIR evaluation of *p*-CoA and *p*-CoA loaded hydrogel.

***
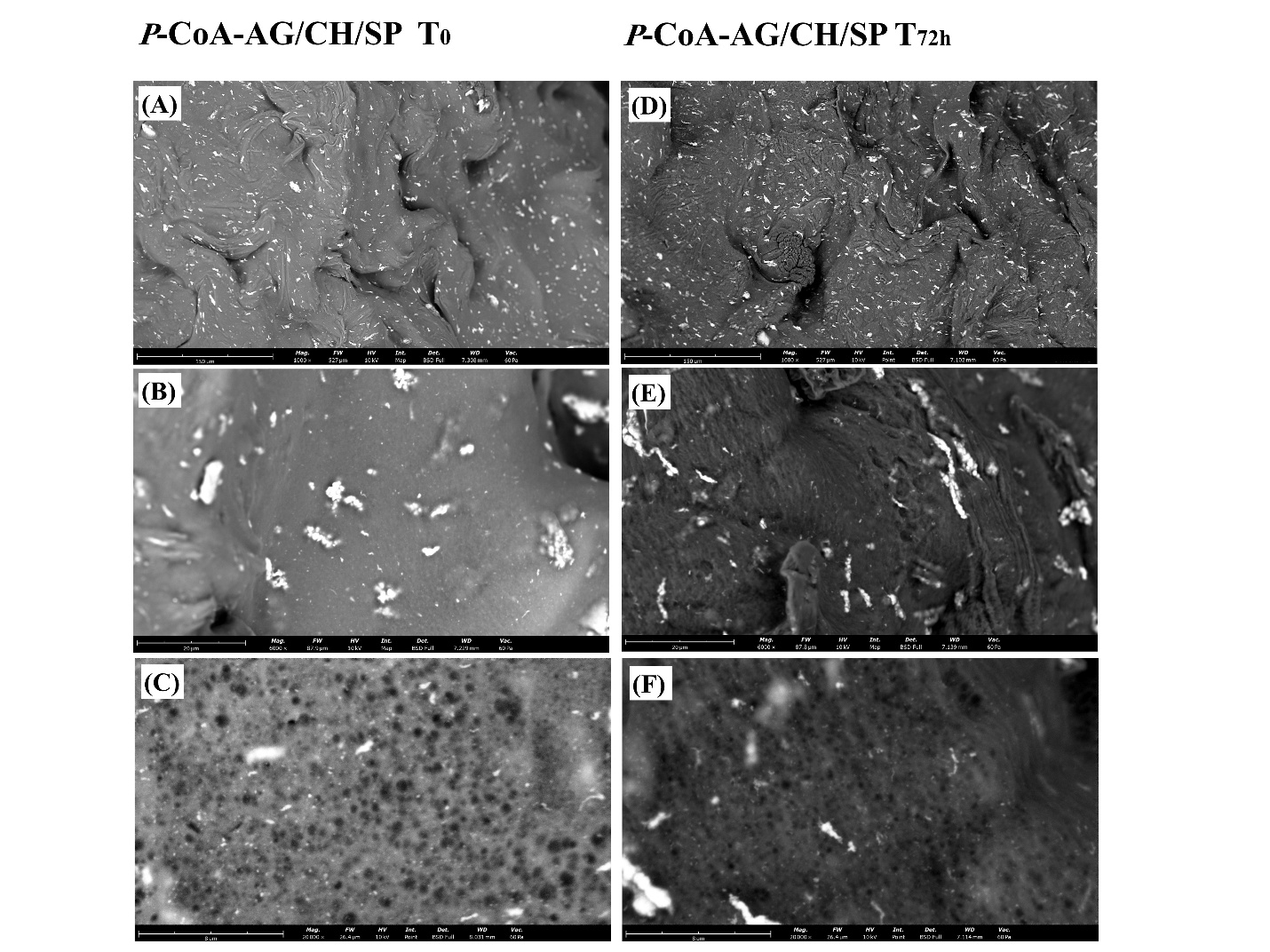
***

**Figure S9.** SEM images of dried sample of *P*-CoA-loaded AG/CH/SP hydrogel at various magnifications: (A) 1000x (B) 6000x (C) 20000x, (D-F): dried *P*-CoA-loaded AG/CH/SP hydrogel after incubation with SGF for 72 h at various magnifications: (D) 1000 (E) 6000 (F) 20000 exhibiting retention of SPIONs in the hydrogel matrix. SPIONs are visible as white spots in the images.
